# Inflammatory and Cytotoxic Activities of Abietane Terpenoids from *Nepeta bracteata* Benth.

**DOI:** 10.3390/molecules26185603

**Published:** 2021-09-15

**Authors:** Manli Zhang, Meiying Chen, Yong Hou, Congzhao Fan, Hongyan Wei, Leiling Shi, Guoxu Ma, Jing Zhang

**Affiliations:** 1 College of Chinese Medicine Material, Jilin Agricultural University, Changchun 130118, China; zmlfl1221@163.com; 2 Key Laboratory of Bioactive Substances and Resource Utilization of Chinese Herbal Medicine, Institute of Medicinal Plant Development, Peking Union Medical College and Chinese Academy of Medical Sciences, Beijing 100193, China; myc1091@163.com (M.C.); houyong@implad.ac.cn (Y.H.); mgxfl8785@163.com (G.M.); 3 Xinjiang Institute of Chinese and Ethnic Medicine, Urumqi 830002, China; fcz_840701@163.com (C.F.); whywlmq@163.com (H.W.)

**Keywords:** terpenoids, *Nepeta bracteata* Benth., anti-inflammatory

## Abstract

*Nepeta bracteata* Benth. is used clinically to treat tracheal inflammation, coughs, asthma, colds, fevers, adverse urination, and other symptoms, along with functions in clearing heat and removing dampness. However, there have been few studies characterizing the material basis of its efficacy. Therefore, the aim of this study was to screen for compounds with anti-inflammatory activities in *N. bracteata* Benth. Using silica gel, ODS C18, and Sephadex LH-20 column chromatography, as well as semipreparative HPLC, 10 compounds were separated from *N. bracteata* Benth. extract, including four new diterpenoids (**1**–**4**), one amide alkaloid (**5**), and five known diterpenoids (**6**–**10**). The structures of all the isolates were elucidated by HR-ESI-MS, NMR, and CD analyses. Using lipopolysaccharide (LPS)-stimulated RAW 264.7 cells, we investigated the anti-inflammatory activities of compounds **1**–**10**. It is worth noting that all were able to inhibit nitric oxide (NO) production with IC_50_ values < 50 μM and little effect on RAW 264.7 macrophage viability. Compounds **2** and **4** displayed remarkable inhibition with IC_50_ values of 19.2 and 18.8 μM, respectively. Meanwhile, screening on HCT-8 cells demonstrated that compounds **2** and **4** also had moderate cytotoxic activities with IC_50_ values of 36.3 and 41.4 μM, respectively, which is related to their anti-inflammatory effects.

## 1. Introduction

**Figure 1:**
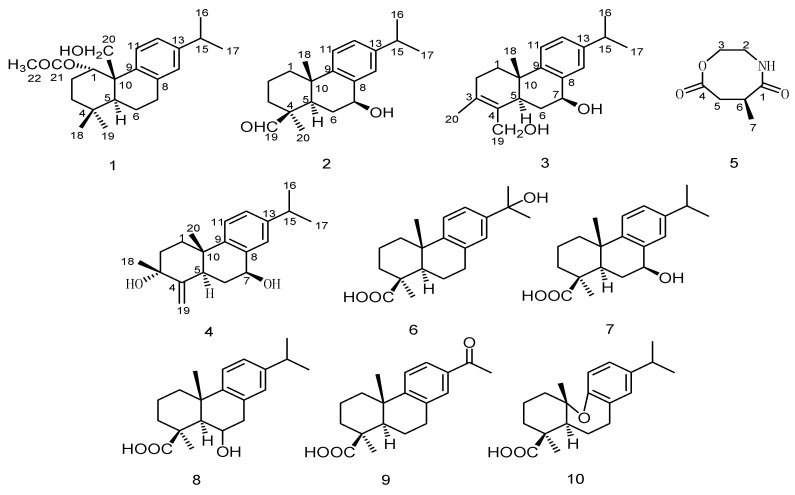
Structures of compounds **1**–**10**.

Inflammation, a common clinical pathological process, is closely related to many diseases such as arthritis, psychosis, cardiovascular and cerebrovascular diseases, and cancer [1,2,3,4]. Current anti-inflammatory drugs, such as glucocorticoids, insulin, and the tyrosinase inhibitor kojic acid, are associated with significant side effects [5,6]. Therefore, finding effective anti-inflammatory drugs with fewer side effects is of great importance. Natural products are an important source for new drug discovery; thus, finding and discovering active components from medicinal plants is a hot topic in pharmaceutical chemistry. *Nepeta bracteata* Benth. belongs to the genus *Nepeta* of the Lamiaceae family and is mainly distributed in Pakistan, Nepal, Iran, and other countries. It is a folk medicine used by Xinjiang Uyghurs and a medicinal material imported for use in the Xinjiang Uygur hospital, with the Uyghur name “Zufa” [7]. Clinically, it is used to treat tracheal inflammation, coughs, asthma, colds, fevers, adverse urination, and other symptoms [8,9], along with functions in clearing heat and removing dampness. While modern pharmacology has shown that its extract has significant anti-inflammatory activity, no related research has been conducted to characterize its chemical components [10,11,12]. With the aim of screening for anti-inflammatory active compounds in *N. bracteata* Benth., a 95% ethanol extract was investigated, and 10 compounds—four new diterpenoids, nepetabrates A–D (1–4), one amide alkaloid, 6-methyl-1,4-oxazocane-5,8-dione (5), and five known diterpenoids, angustanoic acid F (6) [13], 7a-hydroxycallitrisic acid (7) [14], 1-phenanthrenecarboxylic acid (8) [15], angustanoic acid G (9) [16], and jiadifenoic acid K (10) [17]—were obtained (drawn in Figure 1). The structures of the isolates were characterized using comprehensive spectroscopic data analyses. Moreover, the anti-inflammatory activities of the isolated compounds were investigated. In this paper, we describe the structural elucidation of the isolated compounds, as well as their potential anti-inflammatory and cytotoxic effects.

## 2. Results

### 2.1. Structure Elucidation

**Figure 2:**
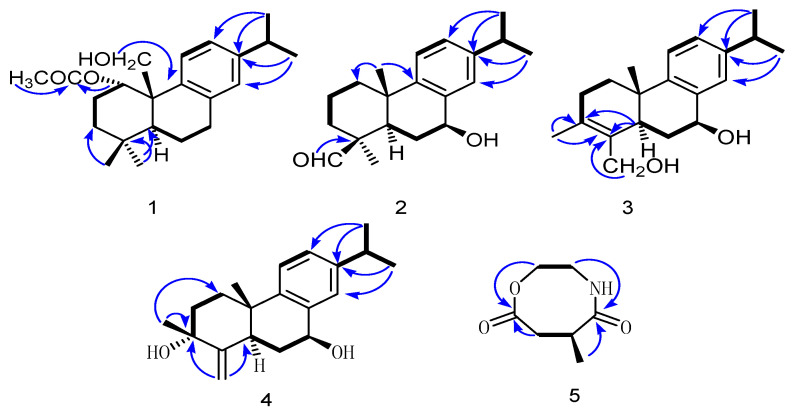
Key ^1^H–^1^H COSY (in bolds) and HMBC (arrows) correlations of compounds **1**–**5**.

**Figure 3:**
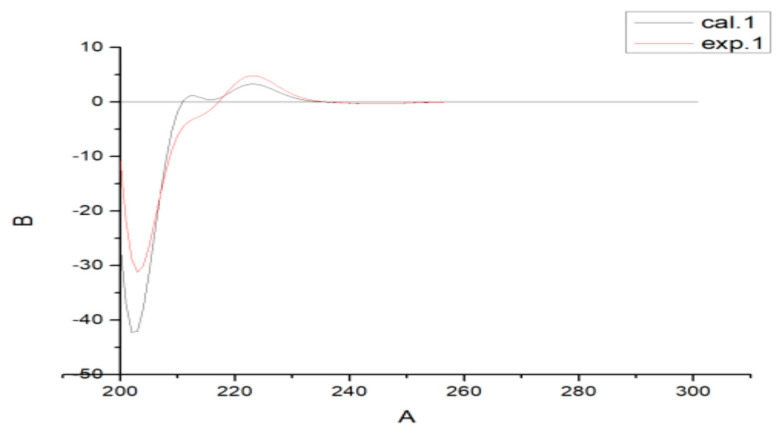
Experimental and calculated ECD spectra of **1**.

Compound **1** was isolated as a white powder. The molecular formula C_22_H_3__2_O_3_ was established from the HR-ESI-MS spectrum with a positive-ion peak at *m*/*z* [M + Na]^+^ 367.2215 (calculated 367.2249). The IR spectrum of **1** showed absorptions of hydroxyl (3339 cm^−1^) and ester carbonyl (1759 cm^−1^) groups. In the ^1^H-NMR spectrum (Table 1), compound **1** showed peaks for three aromatic protons at *δ*_H_ 7.14 (1H, d, *J* = 8.4 Hz), 7.17 (1H, d, *J* = 8.4 Hz), and 7.22 (1H, d, *J* = 1.8 Hz), which suggests the presence of a benzene moiety. Four methyl protons at *δ*_H_ 1.25 (3H, d, *J* = 7.2 Hz), 0.97 (3H, d, *J* = 7.2 Hz), 1.15 (3H, s), and 1.07 (1H, s) indicate the basic diterpenoid skeleton. The downfield methyl signal at *δ*_H_ 2.07 (3H, s) is evidence for the existence of an acetoxyl group. Meanwhile, three oxygenated protons at *δ*_H_ 4.82 (1H, m), 4.00 (1H, d, *J* = 6.6 Hz), and 4.32 (1H, dd, *J* = 6.6, 2.4 Hz) suggest the presence of –OCH_2_– and –OCH– groups in the structure, which is in accordance with the ^13^C-NMR spectrum showing signals at *δ*_C_ 68.5, 67.3. The ^13^C-NMR spectrum (Table 1) revealed six aromatic carbon signals at *δ*_C_ 135.9, 147.1, 124.9, 126.9, 146.8, and 127.9. Aside from these aromatic carbons, the ^1^^3^C-NMR also showed five methyl signals at *δ*_C_ 24.2, 13.9, 24.8, 28.7, and 19.4, five methylene signals at *δ*_C_ 36.2, 37.9, 19.4, 27.4, and 67.3, three methine signals at *δ*_C_ 68.5, 45.3, and 33.7, and two quartus carbon signals at *δ*_C_ 36.8 and 38.6. The proton signals were assigned to the corresponding carbons through direct ^1^H and ^13^C correlations in the HSQC spectrum. From the ^1^H–^1^H COSY analysis, four substructures (drawn with bold bonds in Figure 2) were established as H_2_-1/H_2_-2/H_2_-3, H-5/H_2_-6/H_2_-7, H-11/H-12, and H-15/H_3_-16/H_3_-17, suggesting that compound **1** has an abietane diterpene skeleton [16]. In the HMBC spectrum (Figure 2), the correlations from *δ*_H_ 4.82 (1H, m, H-1) to *δ*_C_ 171.6 indicate that the acetoxyl group is attached to C-1. Meanwhile, the HMBC correlations from *δ*_H_ 4.00 (1H, d, *J* = 6.6 Hz) and 4.32 (1H, dd, *J* = 6.6, 2.4 Hz) to C-10 (*δ*_C_ 38.6) and C-9 (*δ*_C_ 147.1) indicate that the angular methyl group at C-10 was further oxidized to a hydroxylmethyl unit. Thus, the planar structure of compound **1** was fully elucidated. In the NOESY spectrum, the enhancement between H-1 and H_2_-20, H-5, and H_3_-19 suggests the *α*-orientation of the acetoxyl group at C-1. Considering the identical biosynthetic relationship of abietane diterpenoids, the absolute configuration of **1** can be inferred as 1*S*, 5*S*, 10*S*. The ECD spectra were calculated using density functional theory (DFT) at the APFD/6-311 + g (2d, p) level to further support the deduction (Figure 3). As a result, the structure of compound **1** was determined as shown and given the trivial name nepetabrate A.

Compound **2** was obtained as a white powder with its molecular formula assigned as C_20_H_28_O_2_ according to the positive HR-ESI-MS peak at *m*/*z* [M + Na]^+^ 323.2013 (calculated 323.2089), exhibiting seven degrees of unsaturation. Through the ^1^H- and ^13^C-NMR spectra, we inferred that the basic mother nucleus of compound **2** was an abietane diterpene, which was further confirmed by the ^1^H–^1^H COSY and HMBC spectra (Figure 2). In fact, the NMR data of compound **2** were similar to those reported for 1-phenanthrenecarboxylic acid, except for an additional aldehyde signal at *δ*_H_ 9.43 (s), *δ*_C_ 206.6, and oxygenated methine carbon at *δ*_C_ 71.9 [15]. The presence of the aldehyde group was due to the oxidation of a methyl group at C-19, as supported by the correlations between H-19 (*δ*_H_ 9.43) and C-4 (*δ*_C_ 55.5) in the HMBC spectrum. In addition, one hydroxyl group placed at C-7 led to the downfield chemical shift of C-7 (*δ*_C_ 71.9), as confirmed by HMBC correlations. The relative configuration of compound **2** was established by analysis of its NOESY data. The key NOE correlations between H-5 and H-7 and between H_3_-18 and H-19 supported the β-orientations of both the aldehyde and the hydroxyl groups. Combined with the experimental and calculated CD curves (Supplementary Materials. Figure S31), the absolute configuration of compound **2** was identified as established and given the trivial name nepetabrate B.

Compound **3**, purified as a white powder, has a molecular formula of C_20_H_28_O_2_, deduced from the HR-ESI-MS quasimolecular ion at *m*/*z* 323.2021 [M + Na]^+^ (calculated 323.2089). The ^1^H- and ^13^C-NMR spectroscopic data (Table 1) of **3** were similar to those of compound **2**, except for the additional hydroxymethyl group at *δ*_C_ 61.9 and double bond at *δ*_C_ 126.5 and 136.2, indicating that compound **3** is an analogue of compound **2**. In the HMBC spectrum, the correlations from H-5 (*δ*_H_ 2.32) to C-3 (*δ*_C_ 126.5) and C-4 (*δ*_C_ 136.2), from H_3_-20 (*δ*_H_ 1.65) to C-4 (*δ*_C_ 136.2), and from H_2_-19 to C-3 (*δ*_C_ 126.5) (Figure 2) implied a double bond at C-3/C-4, as well as substitutions of its methyl and hydroxymethyl groups. Compound **3** is the product of methyl migration and dehydrogenation of compound **2**, which is not common in abietane diterpenes. The similar NOESY spectra of compounds **2** and **3** suggest their identical relative configurations. Together with the experimental and calculated CD curves (Supplementary Materials, Figure S32), compound **3** was elucidated as nepetabrate C.

Compound **4** was obtained as a white powder with a molecular formula of C_20_H_28_O_2_ based on the positive-ion HR-ESI-MS peak at *m/z* 323.2023 [M + Na]^+^ (calculated 323.2089). The ^1^H- and ^13^C-NMR spectroscopic data (Table 1) of **4** were quite similar to those of **3**, except for the appearance of olefin protons at *δ*_H_ 5.12 and 4.74 and the disappearance of two oxygenated protons in **4**. Further analysis of the NMR data of compound **4** revealed the presence of an outer ring double bond at C-4/19, which was confirmed by the HMBC correlations from H-19 (*δ*_H_ 4.78, 4.69) to C-3 (*δ*_C_ 71.5) and C-5 (*δ*_C_ 36.9). Furthermore, the quartus carbon of C-3 (*δ*_C_ 71.5), together with the molecular formula above, implies the presence of hydroxyl substitution in the structure. The HMBC correlations from H_3_-18 (*δ*_H_ 0.88) to *δ*_C_ 71.5 reveal the location of CH_3_-18 at C-3. The key NOESY enhancements between H-5 and H-7 and between CH_3_-18 and CH_3_-20 revealed the opposing configurations of 3-OH and 7-OH. The absolute configuration was determined by DFT calculations of the ECD spectra (Supplementary Materials, Figure S33), which confirmed the 3*R*, 5*R*, 7*S*, and 10*S* configurations. Thus, compound **4** was assigned as nepetabrate D.

Compound **5** was obtained as a yellow amorphous powder, with its molecular formula assigned as C_7_H_11_NO_3_ on the basis of its positive HR-ESI-MS (*m/z* 180.0723 [M + Na]^+^ (calculated 180.0739), implying three degrees of unsaturation. The IR spectrum of **5** showed carbonyl (1724 cm^−1^) and methyl (2938, 2924 cm^−1^) groups. In the ^1^H-NMR spectrum (Table 1), compound **5** showed four downfield chemical signals at *δ*_H_ 3.79, 2.97, 2.99, and 2.39, one methyl proton at *δ*_H_ 1.37 (3H, d, *J* = 7.2 Hz), and one methine proton at *δ*_H_ 2.91 (1H, m). The ^13^C-NMR spectrum (Table 1) revealed the presence of two carbonyl groups at *δ*_C_ 177.4 and 181.5, three methylene carbons at *δ*_C_ 41.9, 60.9, and 36.6, one methine carbon at *δ*_C_ 34.9, and one methyl carbon at *δ*_C_ 16.9. Analysis of the ^1^H–^1^H COSY correlations revealed the presence of two partial structures of –(CH_2_)_2_– and –CH_2_–(CH)CH_3_–, as shown in Figure 2. In the HMBC spectrum, the correlations from *δ*_H_ 3.79, 2.97 (2H, m, H-3) and *δ*_H_ 2.99 (1H, H-5a), 2.39 (1H, dd, *J* = 4.2, 4.2 Hz, H-5b) to *δ*_C_ 181.5 (C-4), *δ*_H_ 3.71, 2.87 (2H, m, H-2), and *δ*_H_ 1.37 (3H, d, *J* = 7.2 Hz, H_3_-7) to *δ*_C_ 177.4 (C-1) demonstrate that the two fragments were connected through ester carbonyl carbons. Further analysis of the carbon signal of C-2 (*δ*_C_ 41.9) and the molecular formula above confirmed an amide unit between C-2 and C-6. The absolute configuration of C-6 was assigned as *S* on the basis of a comparison of its experimental and calculated CD curves. From all the above data, compound **5** was established as shown and named 6-methyl-1,4-oxazocane-5,8-dione.

### 2.2. Bioactive Activity

The anti-inflammatory activities of compounds **1**–**10** were evaluated in RAW 264.7 macrophages with aspirin as the positive control [18,19]. As shown in Table 2, in the RAW 264.7 macrophage viability test, all compounds showed mild or inavtive effects for RAW 264.7 macrophages. On the contrary, compounds **1**–**10** displayed different degrees of anti-inflammatory activity against RAW 264.7 macrophages, with IC_50_ values ranging from 18.0 to 46.3 μM. Among the abietane diterpenes, compounds **2** and **4** displayed the most anti-inflammatory activity with IC_50_ values of 19.2 and 18.8 μM, respectively. Compound **5**, as an amide alkaloid, also showed a remarkable inhibition effect with an IC_50_ value of 18.0 μM, as compared with the IC_50_ values of 15.9 for the positive control, aspirin. Cytotoxic testing on HCT-8 cells showed that compounds **2** and **4** had moderate activity with IC_50_ values of 36.3 and 41.4 μM, respectively, while the other compounds were inactive or mildly active when compared with the positive control, adriamycin.

## 3. Discussion

Although *Nepeta bracteata* Benth. is widely used clinically and has promising curative effects, there are few studies about this medicinal plant. Previous research found that *N. bracteata* Benth. has certain free-radical-scavenging ability and an anti-inflammatory effect in vitro, and its alcohol extract displayed certain DPPH free-radical-scavenging ability, providing a theoretical basis for further research [20,21]. Therefore, we first investigated the active substances of *N. bracteate* Benth. and obtained nine abietane diterpenoids including four new ones, nepetabrates A–D (1–4), and one new amide alkaloid (5). Compared with previous studies, we explored the anti-inflammatory activities of compounds 1–10 on RAW 264.7 macrophages cells. The cell viability of the RAW 264.7 macrophages experiment displayed that all the isolated compounds had mild toxicity to cells at 50 μM. The anti-inflammatory activity test showed that all the abietane diterpenoids displayed different degrees of inhibition effect. Among them, compounds 2 and 4 displayed the greatest anti-inflammatory activities with IC_50_ values 19.2 and 18.8 μM, as well as moderate cytotoxic activities with IC_50_ values of 36.3 and 41.4 μM, further proving the correlation between inflammation and cancer. Morever, the results also showed that compound 5 had significant anti-inflammatory activity but had no significant advantage over diterpenes. Accordingly, we believe that diterpenes represent the material basis for the plant to exert its clinical anti-inflammatory effect, which deserves further study.

## 4. Materials and Methods

### 4.1. General Experimental Procedures

Optical rotation data were measured using a Perkin-Elmer 341 digital polarimeter (PerkinElmer, Norwalk, OH, USA). UV (1.0 mg of sample was dissolved in 3 mL of chromatographic grade methanol for each sample) and IR (1.0 mg of sample was pressed in KBr for each sample) spectral data were recorded on Shimadzu UV2550 and FTIR-8400S spectrometers (Shimadzu, Kyoto, Japan). CD spectra were obtained using a JASCO J-815 spectropolarimeter. NMR spectra were obtained using a Bruker AV III 600 NMR spectrometer with chemical shift values presented as δ values using TMS as the internal standard (samples dissolved in an appropriate amount ofdeuterated chloroform). HR-ESI-MS was performed using an LTQ-Orbitrap XL spectrometer (Thermo Fisher Scientific, Boston, MA, USA); samples were dissolved in chromatographic methanol and treated through a membrane, single pump. Colurmn chromatography (CC) was performed using silica gel (100–200 and 200–300 mesh, Qingdao Marine Chemical Plant, Qingdao, China). Semi-preparative HPLC was performed using an HPLC PUMP K-501, LC3000 high-performance liquid chromatograph (Beijing Tong Heng Innovation Technology Co., Ltd, Beijing, China), and Kromasil 100-5C18, 250 × 10 mm, E108850. Precoated silica gel GF_254_ plates (Zhi Fu Huang Wu Pilot Plant of Silica Gel Development, Yantai, China) were used for TLC. All solvents used (petroleum ether, ethyl acetate, dichloromethane, methanol (analytical grade and chromatographic grade), and deuterated chloroform) were of analytical grade (Beijing Chemical Plant, Beijing, China).

### 4.2. Plant Material

*Nepeta bracteata* Benth. was purchased from Xinjiang Uygur hospital (Urumqi, China) and identified as *Nepeta bracteata* Benth. by Professor Leiling Shi. A voucher specimen (M20191025) was deposited at the Medical Laboratory of Xinjiang Institute of Chinese and Ethnic Medicine (Urumqi, China).

### 4.3. Isolation and Purification of Compounds ***1***–***10***

The aerial part of *Nepeta bracteata* Benth. (6.0 kg) was soaked in ethanol at room temperature (3 × 40 L, 3 h each time) and extracted three times under reflux. Removal of the ethanol under reduced pressure yielded the ethanol extract (437.0 g). The ethanol extract was dissolved in water and successively extracted with petroleum ether (3 × 1000 mL), dichloromethane (3 × 1000 mL), and ethyl acetate (3 × 1000 mL). The petroleum fraction (134.8 g) was subjected to CC (12.0 cm × 40.0 cm, 300.0 g) over a silica gel (100–200 mesh), eluting with a stepwise gradient of petroleum ether/EtOAC (from 1:0 to 0:1; i.e., 1:0, 100:1, 50:1, 25:1, 8:1, 5:1, 1:1, and 0:1, *v*/*v*) to yield fractions A–H. Fr.F was subjected to CC (5.0 cm × 15.0 cm, 70.0 g) over a silica gel (100–200 mesh), eluting with a stepwise gradient of petroleum ether/EtOAC (from 20:1 to 1:1; i.e., 20:1, 10:1, 3:1, and 1:1, *v*/*v*) to yield four fractions (Fr.F 1–4). Fr.F 3 was subjected to CC (3.0 cm × 20.0 cm, 65.0 g) over a silica gel (200–300 mesh), eluting with a stepwise gradient of petroleum ether/EtOAC (from 10:1 to 3:1; i.e., 10:1, 5:1, and 3:1, *v*/*v*) to yield three fractions (Fr.F 3-1–3). Fr.F 3-2 was purified using semi-preparative HPLC with MeOH/H_2_O (90:10, *v*/*v*) as the mobile phase to yield compound **1** (9.0 mg, t_R_ = 42.4 min). Fr.F 3-3 was purified using semi-preparative HPLC of MeOH/H_2_O (85:15, *v*/*v*) as the mobile phase to yield compounds **2** (5.2 mg, t_R_ = 27.0 min) and **3** (9.2 mg, t_R_ = 28.6 min). Fr.H was subjected to CC (4.0 cm × 15.0 cm, 30.0 g) over a silica gel (100–200 mesh), eluting with a stepwise gradient of petroleum ether/EtOAC (from 15:1 to 1:1; i.e., 15:1, 5:1, 3:1 and 1:1, *v*/*v*) to yield four fractions ( Fr.H 1–4 ). Fr.F 3 was subjected to CC (3.0 cm × 20.0 cm, 70.0 g) over a silica gel (200–300 mesh), eluting with a stepwise gradient of petroleum ether/EtOAC (from 4:1 to 1:1; i.e., 4:1, 2:1, and 1:1, *v*/*v*) to yield three fractions (Fr.H 3-1–3). Fr.F 3-3 was purified by semi-preparative HPLC of MeOH/H_2_O (85:15, *v*/*v*) as the mobile phase to yield compound **4** (3.2 mg, t_R_ = 25.6 min). The dichloromethane fraction (67.4 g) was subjected to CC (8.0 cm × 40.0 cm, 240.0 g) over a silica gel (100–200 mesh), eluting with a stepwise gradient of CH_2_Cl_2_/MeOH (from 1:0 to 0:1; i.e., 1:0, 100:1, 50:1, 30:1, 20:1, 5:1, 1:1, and 0:1, *v*/*v*) to yield fractions I–O. Fr.I was subjected to CC (8.0 cm × 15.0 cm, 105.0 g) over a silica gel (100–200 mesh), eluting with a stepwise gradient of petroleum ether/EtOAC (from 15:1 to 1:2; i.e., 15:1, 8:1, 4:1, 2:1, 1:1, and 1:2, *v*/*v*) to yield six fractions (Fr.I 1–6). Fr.I 3 was isolated through ODS MPLC elution with MeOH/H_2_O (50:50, 70:30, 90:10, and 100:0, *v*/*v*), and purified using semi-preparative HPLC to give compounds **6** (2.0 mg, t_R_ = 23.2 min), **7** (2.3 mg, t_R_ = 26.4 min), and **8** (1.4 mg, t_R_ = 12.7 min). Fr.J was subjected to CC (6.0 cm × 20.0 cm, 70.0 g) over a silica gel (100–200 mesh), eluting with a stepwise gradient of petroleum ether/EtOAC (from 15:1 to 1:2; i.e., 15:1, 8:1, 4:1, 2:1, 1:1, and 1:2 *v*/*v*) to yield six fractions (Fr.J 1–6). Fr.J 2 was purified by semi-preparative HPLC of MeOH/H_2_O (85:15, *v*/*v*) as the mobile phase to yield compounds **9** (3.6 mg, t_R_ = 15.9 min) and **10** (1.4 mg, t_R_ = 19.3 min). Fr.J 6 was purified by semi-preparative HPLC of MeOH/H_2_O (38:62, *v*/*v*) as the mobile phase to yield compound **5** (3.6 mg, t_R_ = 9.5 min). 

### 4.4. Characterization of Compounds ***1***–***5***

Nepetabrate A (**1**), white powder (MeOH); UV (MeOH) λ_max_ (logε) 291 (3.52) nm; IR (film) ν_max_ 3339, 2962, 2871, 1759, 1467, 1213, 1144 cm^–1^; ^1^H- and ^13^C-NMR data (CDCl_3_), see Table 1; HR-ESI-MS *m*/*z* 367.2215 [M + Na]^+^ (calculated 367.2249, C_22_H_32_O_3_).

Nepetabrate B (**2**), white powder (MeOH); UV (MeOH) λ_max_ (logε) 294 (3.76) nm; IR (film) ν_max_ 3338, 2994, 2936, 2872, 1460, 1221, 1099 cm^–1^; ^1^H- and ^13^C-NMR data (CDCl_3_), see Table 1; HR-ESI-MS *m*/*z* 323.2013 [M + Na]^+^ (calculated 323.2089, C_20_H_28_O_2_).

Nepetabrate C (**3**), white powder (MeOH); UV (MeOH) λ_max_ (logε) 292 (3.93) nm; IR (film) ν_max_ 3342, 2958, 2875, 1456, 1230, 1138 cm^–1^; ^1^H- and ^13^C-NMR data (CDCl_3_), see Table 1; HR-ESI-MS *m*/*z* 323.2021 [M + Na]^+^ (calculated 323.2089, C_20_H_28_O_2_).

Nepetabrate D (**4**), white powder (MeOH); UV (MeOH) λ_max_ (logε) 294 (3.53) nm; IR (film) ν_max_ 3351, 2940, 2866, 1445, 1227, 1115 cm^–1^; ^1^H- and ^13^C-NMR data (CDCl_3_), see Table 1; HR-ESI-MS *m*/*z* 323.2023 [M + Na]^+^ (calculated 323.2089, C_20_H_28_O_2_).

6-Methyl-1,4-oxazocane-5,8-dione (**5**), yellow amorphous powder (MeOH); UV (MeOH) λ_max_ (logε) 291 (3.76) nm; IR (film) ν_max_ 3350, 2938, 2874, 1724 cm^–1^; ^1^H- and ^13^C-NMR data (CDCl_3_), see Table 1; HR-ESI-MS *m*/*z* 180.0723 [M + Na]^+^ (calculated 180.0739, C_7_H_11_NO_3_).

### 4.5. RAW 264.7 Macrophage Viability Test

The MTT colorimetric method was used to detect the effect of compounds **1**–**10** on the viability of RAW 264.7 macrophages. The RAW 264. 7 macrophages in the logarithmic growth phase were digested with trypsin to prepare a single-cell suspension, which was seeded in a 96-well plate at a density of 1 × 10^4^ cells per well and cultured in a 5% CO_2_ incubator for 24 h at 37 °C, before discarding the supernatant. The blank control group was cultured with 10% FBS-containing DMEM, and the drug group was treated with aqueous solutions of compounds **1**–**10**, with six replicate wells for each concentration. Incubation was continued in 5% CO_2_ at 37 °C. After 24 h of incubation, 10 μL of 5 mg/mL MTT was added to each well. The culture solution was removed after culturing for 4 h. Then, 100 μL of DMSO was added to each well, before shaking for 10 min to achieve complete dissolution. The optical density (OD) was measured at 492 nm using a microplate reader to calculate cell viability.

### 4.6. Anti-Inflammation Assay

The anti-inflammatory activity of the isolated compounds was evaluated in lipopolysaccharide-stimulated RAW 264.7 macrophages using the MTT colorimetric method. The RAW 264.7 macrophages were seeded in 96-well plates at a density of 1 × 10^4^ cells per well for 24 h, followed by treatment with different extracts of identical purity for another 24 h. The compounds were dissolved in dimethyl sulfoxide (DMSO) and diluted appropriately just before cell treatments. Cells were incubated with the extract at indicated concentrations, with DMSO not exceeding 0.1% in all experiments. The cells were cultured in DMEM with 10% FBS and antibiotics (100 U/mL penicillin and 100 µg/mL streptomycin) at 37 °C with 5% CO_2_. NO release was measured as an indicator of the nitrite concentration.

### 4.7. Cytotoxicity Test

The cytotoxic activities of compounds **1**–**10** against HCT-8 cells were tested using the MTT colorimetric method. HCT-8 cells were cultivated on DMEM medium at 37 °C and 5% CO_2_. After diluting the DMEM medium, cells were seeded into 96-well sterile microplates (6 × 10^4^ cells/well) and cultured with a series of various concentrations of tested compounds or adriamycin (positive control) for 24 h at 37 °C. After incubation, all compounds were tested at five concentrations (10−100 μM) for 1 h. Following this, the supernatant was removed, and all components were dissolved in 100% DMSO, at such an amount that there was a final DMSO concentration of 0.1% added to each well. The absorbance was measured using a microplate reader at a wavelength of 570 nm. Data are displayed as the means ± SD (n = 3). The cell growth assay was repeated three times, and the IC_50_ values were calculated using Microsoft Excel software. 

## 5. Conclusions

Nine abietane diterpenoids, including four new ones and one new amide alkaloid, were obtained from the ethnic medicine *Nepeta bracteata* Benth. for the first time, which clarified the active substances of *N.* *bracteata* Benth. and laid the foundation for its further clinical application. Furthermore, the anti-inflammatory and cytotoxic activities of all isolates were tested. Compounds **2** and **4** displayed potential biological activities with IC**_50_** values of 19.2 and 18.8 μM in the anti-inflammation assay and IC_50_ values of 36.3 and 41.4 μM in the cytotoxicity test, respectively. Both compounds are active molecules with potential research value.

## Figures and Tables

**Table 1:** NMR spectral data of **1**–**5** (600 MHz for ^1^H-NMR and 150 MHz for ^13^C-NMR).

No.	1 ^a^		2 ^a^		3 ^a^		4 ^a^		5 ^a^	
	*δ*_C_, Type	*δ*_H_ (*J* in Hz)	*δ*_C_, Type	*δ*_H_ (*J* in Hz)	*δ*_C_, Type	*δ*_H_ (*J* in Hz)	*δ*_C_, Type	*δ*_H_ (*J* in Hz)	*δ*_C_, Type	*δ*_H_ (*J* in Hz)
1	68.5, CH	4.82, m	36.2, CH_2_	2.38, m; 1.63, m	34.4, CH_2_	2.33, m; 1.62, m	39.1, CH_2_	1.90, m; 1.65, m	177.4, C	--
2	36.2, CH_2_	1.82, m; 1.21, m	29.9, CH_2_	2.89, m; 1.27, m	30.5, CH_2_	2.76, m; 1.23, m	30.5, CH_2_	1.86, m; 1.29, m	41.9, CH_2_	3.71, m; 2.87, m
3	37.9, CH_2_	2.33, d (12.6);5566771.43, m	21.1, CH_2_	1.89, m; 1.33, m	126.5, C	--	71.5, C	--	60.9, CH_2_	3.79, m; 2.97, m
4	36.8, C	--	55.5, C	--	136.2, C	--	153.7, C	--	181.5, C	--
5	45.3, CH	1.87, m	43.0, CH	1.87, m	37.7, CH	2.32, d (6.0)	36.9, CH	2.31, m	36.6, CH_2_	2.99, m; 2.30, m
6	19.4, CH_2_	1.46, m; 1.72, m	27.2, CH_2_	1.95, m; 1.85, m	32.8, CH_2_	1.75, m; 1.63, m	33.7, CH_2_	2.01, m; 1.03, m	34.9, CH	2.91, m
7	27.4, CH_2_	2.13, m; 2.01, m	71.9, CH	3.85, dd (8.4, 4.2)	69.5, CH	4.83, t (9.0)	68.6, CH	4.86, t (4.8)	16.9, CH_3_	1.37, d (7.2)
8	135.9, C	--	134.6, C	--	136.4, C	--	135.9, C	--	--	--
9	147.1, C	--	146.5, C	--	148.0, C	--	146.9, C	--	--	--
10	38.6, C	--	38.2, C	--	36.5, C	--	43.0, C	--	--	--
11	124.9, CH	7.14, d (8.4)	124.7, CH	7.17, d (9.0)	124.3, CH	7.28, d (7.8)	125.7, CH	7.25, s	--	--
12	126.9, CH	7.17, d (8.4)	127.2, CH	6.91, d (9.0)	126.6, CH	7.16, dd (7.8, 2.4)	126.8, CH	7.15, dd (8.4, 1.8)	--	--
13	146.8, C	--	145.7, C	--	144.5, C	--	144.7, C	--	--	--
14	127.9, CH	7.22, d (1.8)	129.0, CH	7.72, d (3.6)	128.2, CH	7.72, d (2.4)	128.3, CH	7.22, d (8.4)	--	--
15	33.7, CH	2.89, m	33.7, CH	2.83, m	24.4, CH	1.95, m	33.7, CH	1.24, m	--	--
16	24.2, CH_3_	1.25, d (7.2)	13.9, CH_3_	1.21, s	19.4, CH_3_	0.85, s	24.2, CH_3_	1.26, dd (6.6, 2.4)	--	--
17	13.9, CH_3_	0.97, t (7.2)	13.9, CH_3_	1.21, s	21.5, CH_3_	0.85, s	24.0, CH_3_	1.26, dd (6.6, 2.4)	--	--
18	24.8, CH_3_	1.15, s	25.4, CH_3_	1.22, d (1.8)556677	33.7, CH_3_	1.79, s	21.0, CH_3_	0.88, s	--	--
19	28.7, CH_3_	1.07, s	206.6, CH	9.43, s	61.9, CH_2_	4.78, d (12.0);5566774.69, d (12.0)	107.1, CH_2_	5.12, s5566774.74, s	--	--
20	67.3, CH_2_	4.00, d (6.6)5566774.32, dd (6.6,2.4)	23.7, CH_3_	1.16, s	13.9, CH_3_	1.65, s	28.1, CH_3_	1.45, s	--	--
21	171.6, C	--	--	--	--	--	--	--	--	--
22	21.2, CH_3_	2.07, s	--	--	--	--	--	--	--	--

*^a^* Spectraal data were recorded in CDCl_3_.

**Table 2:** Anti-inflammatory and cytotoxic activities of the isolated compounds.

Compounds	Cell Survival Rate (%)	IC_50_ (μM)	
	RAW 264.7 Macrophages	RAW 264.7 Macrophages	HCT-8
**1**	97.93 ± 0.26	38.2 ± 1.15 ^a^	>50
**2**	88.26 ± 0.32	19.2 ± 1.25	36.3 ± 1.10
**3**	99.84 ± 0.21	22.3 ± 1.26	>50
**4**	92.21 ± 0.19	18.8 ± 0.75	41.4 ± 0.91
**5**	99.21 ± 0.15	18.0 ± 1.13	>50
**6**	99.89 ± 0.13	36.2 ± 1.21	>50
**7**	98.75 ± 0.45	37.1 ± 0.81	>50
**8**	99.12 ± 0.28	37.5 ± 0.92	>50
**9**	98.26 ± 0.18	42.3 ± 0.56	>50
**10**	98.75 ± 0.20	46.3 ± 1.02	>50
Aspirin ^b^		15.9 ± 0.38	
Adriamycin			1.78 ± 0.14
DMEM (PBS) ^c^	100.00		

^a^ Values are means ± SD of triplicate experiments. ^b^ Positive control substance. ^c^ Negative control substance.

## Data Availability

The data of the NMR and cellular anti-inflammatory and toxic activity presented in this study are available in supporting information.

## Supplementary Materials

Supplementary data associated with this article can be found in the online version.

## Sample Availability

Samples of the compounds **1**–**10** are available from the authors.
