# Supplementary material for: Inflammatory and Cytotoxic Activities of Abietane Terpenoids from Nepeta bracteata Benth
Source: Molecules. 2021 Sep 15;26(18):5603. doi: 10.3390/molecules26185603 (PMC8466420; doi:10.3390/molecules26185603)
Supplement: Supplementary file 1 [file molecules-26-05603-s001.zip › molecules-1355989-supplementary.pdf]

Supporting Information

Figure S1 ^{1}H-NMR (600 MHz, $CDCL_3$) spectrum of **1** 2
Figure S2 ^{13}C-APT (150 MHz, $CDCL_3$) spectrum of **1** 2
Figure S3 ^{1}H -^{1}H COSY ($CDCL_3$) spectrum of **1** 3
Figure S4 HSQC ($CDCL_3$) spectrum of **1** 3
Figure S5 NOESY ($CDCL_3$) spectrum of **1** 4
Figure S6 HMBC ($CDCL_3$) spectrum of **1** 4
Figure S7 ^{1}H-NMR (600 MHz, $CDCL_3$) spectrum of **2** 5
Figure S8 ^{13}C-APT (150 MHz, $CDCL_3$) spectrum of **2** 5
Figure S9 ^{1}H -^{1}H COSY ($CDCL_3$) spectrum of **2** 6
Figure S10 HSQC ($CDCL_3$) spectrum of **2** 6
Figure S11 NOESY ($CDCL_3$) spectrum of **2** 7
Figure S12 HMBC ($CDCL_3$) spectrum of **2** 7
Figure S13 ^{1}H-NMR (600 MHz, $CDCL_3$) spectrum of **3** 8
Figure S14 ^{13}C-APT (150 MHz, $CDCL_3$) spectrum of **3** 8
Figure S15 ^{1}H -^{1}H COSY ($CDCL_3$) spectrum of **3** 9
Figure S16 HSQC ($CDCL_3$) spectrum of **3** 9
Figure S17 NOESY ($CDCL_3$) spectrum of **3** 10
Figure S18 HMBC ($CDCL_3$) spectrum of **3** 10
Figure S19 ^{1}H-NMR (600 MHz, $CDCL_3$) spectrum of **4** 11
Figure S20 ^{13}C-APT (150 MHz, $CDCL_3$) spectrum of **4** 11
Figure S21 ^{1}H -^{1}H COSY ($CDCL_3$) spectrum of **4** 12
Figure S22 HSQC ($CDCL_3$) spectrum of **4** 12
Figure S23 NOESY ($CDCL_3$) spectrum of **4** 13
Figure S24 HMBC ($CDCL_3$) spectrum of **4** 13
Figure S25 ^{1}H-NMR (600 MHz, $CDCL_3$) spectrum of **5** 14
Figure S26 ^{13}C-APT (150 MHz, $CDCL_3$) spectrum of **5** 14
Figure S27 ^{1}H -^{1}H COSY ($CDCL_3$) spectrum of **5** 15
Figure S28 HSQC ($CDCL_3$) spectrum of **5** 15
Figure S29 NOESY ($CDCL_3$) spectrum of **5** 16
Figure S30 HMBC ($CDCL_3$) spectrum of **5** 16
Figure S31 Experimental and calculated ECD spectra of **2** 17
Figure S32 Experimental and calculated ECD spectra of **3** 17
Figure S33 Experimental and calculated ECD spectra of **4** 18
Figure S34 Experimental and calculated ECD spectra of **5** 18

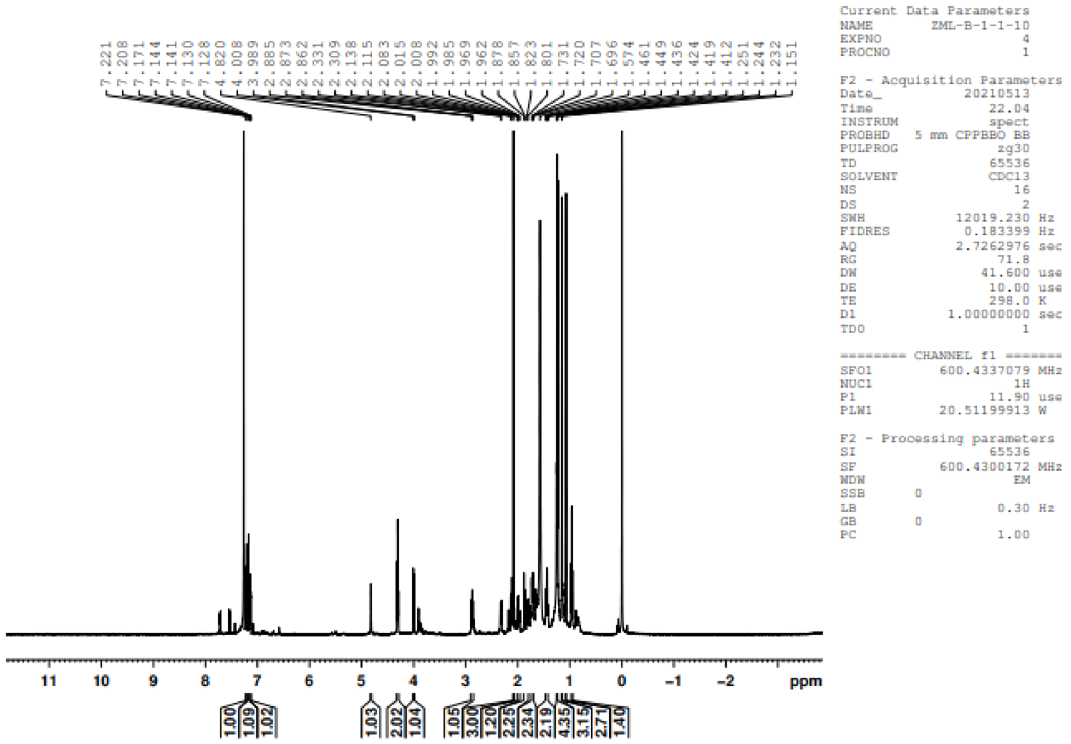

Figure S1 ^{1}H-NMR (600 MHz, CDCL$_3$) spectrum of **1**

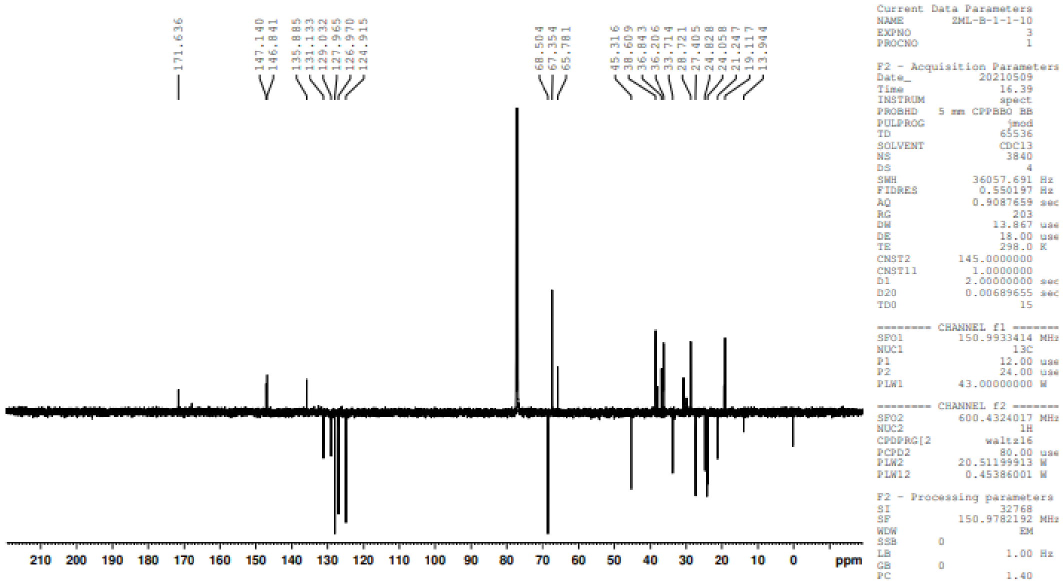

Figure S2 ^{13}C-APT (150 MHz, CDCL$_3$) spectrum of **1**

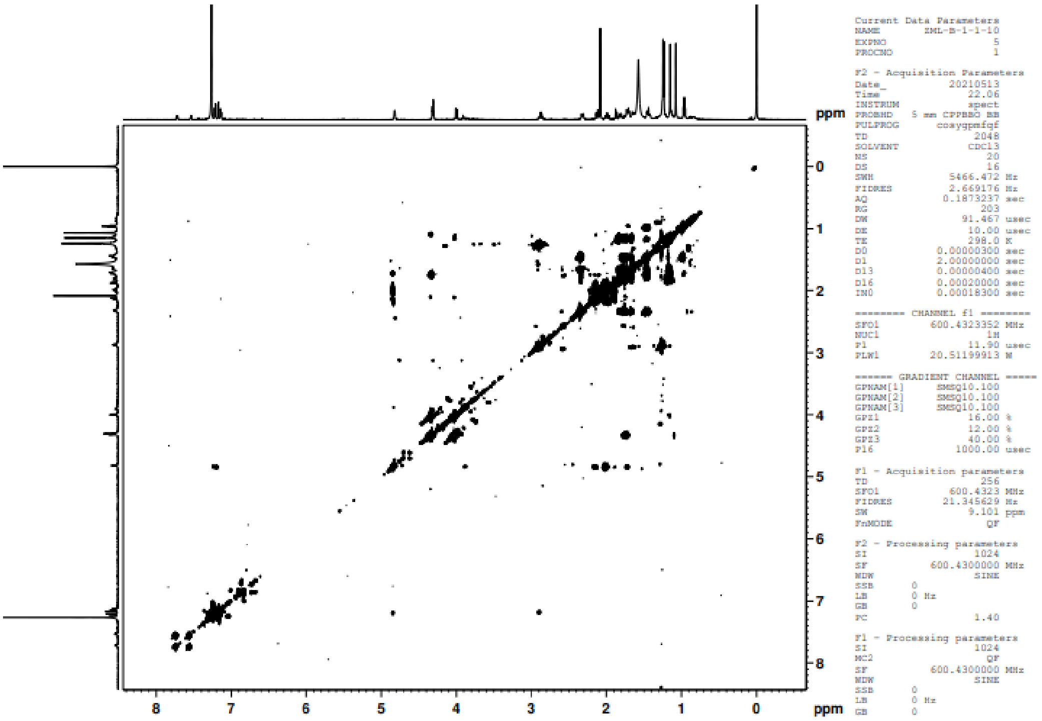

Figure S3 ^{1}H-^{1}H COSY (CDCL$_3$) spectrum of **1**

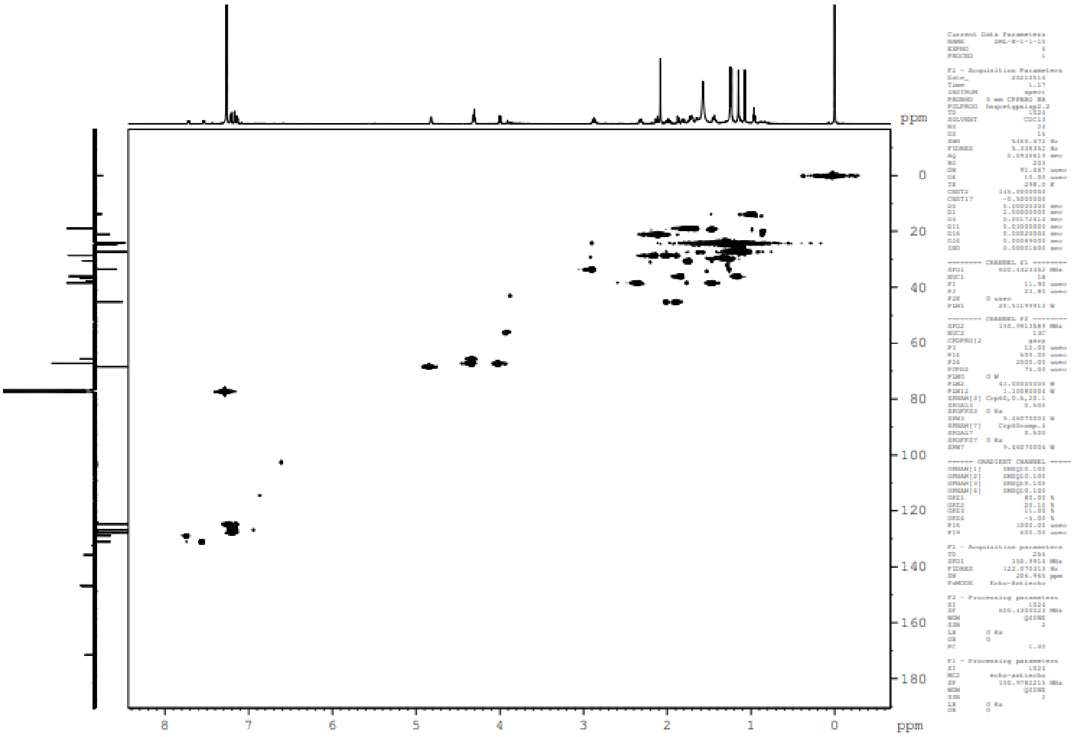

Figure S4 HSQC (CDCL$_3$) spectrum of **1**

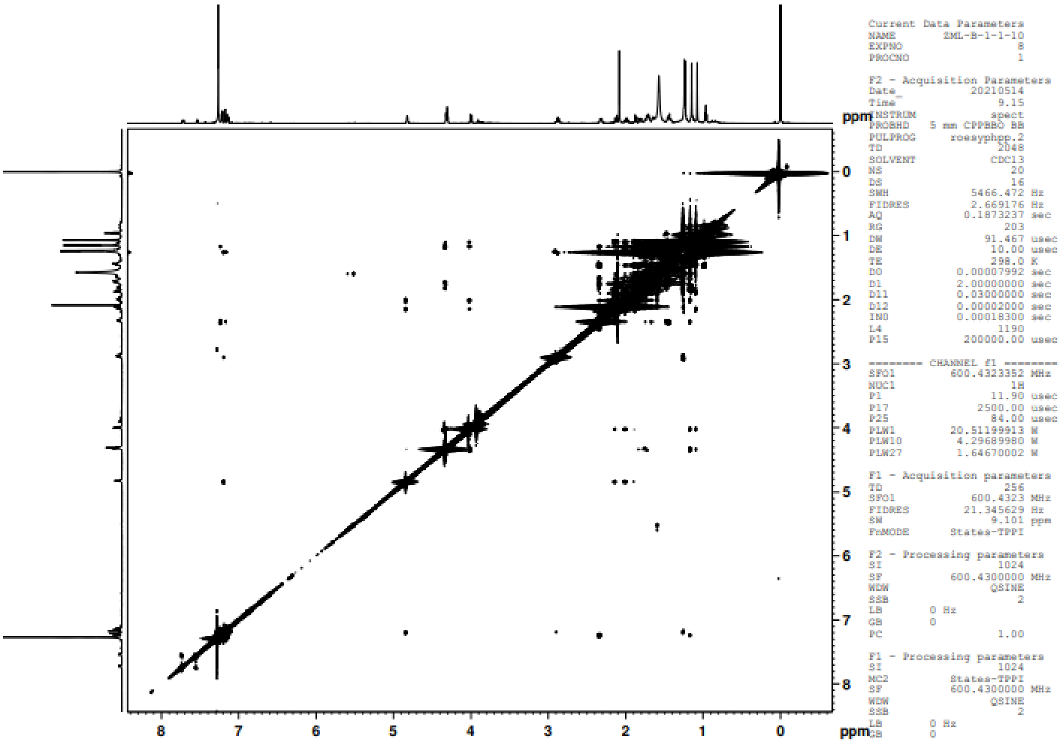

Figure S5 NOESY ($CDCL_3$) spectrum of **1**

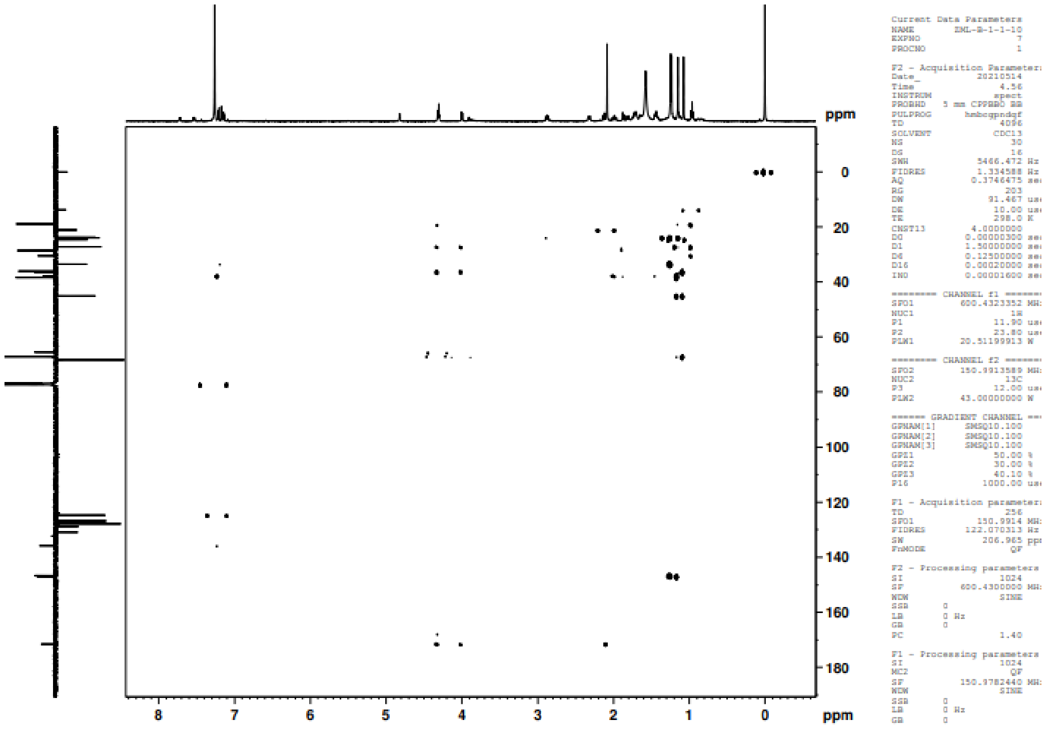

Figure S6 HMBC ($CDCL_3$) spectrum of **1**

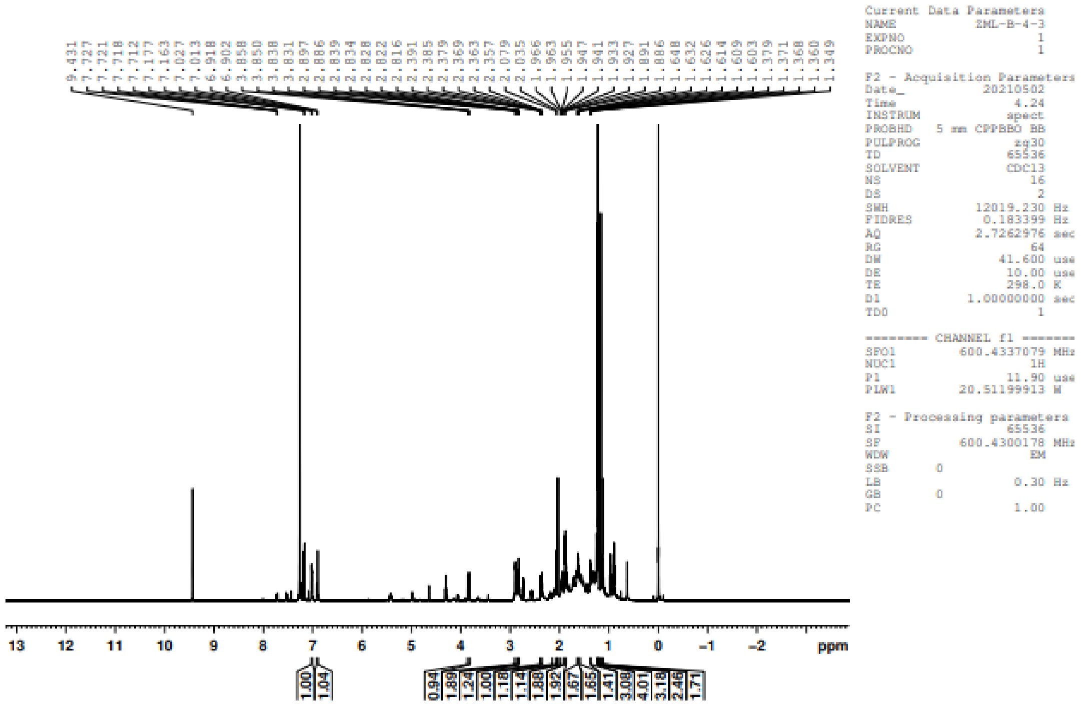

Figure S7 ^{1}H-NMR (600 MHz, $CDCL_3$) spectrum of **2**

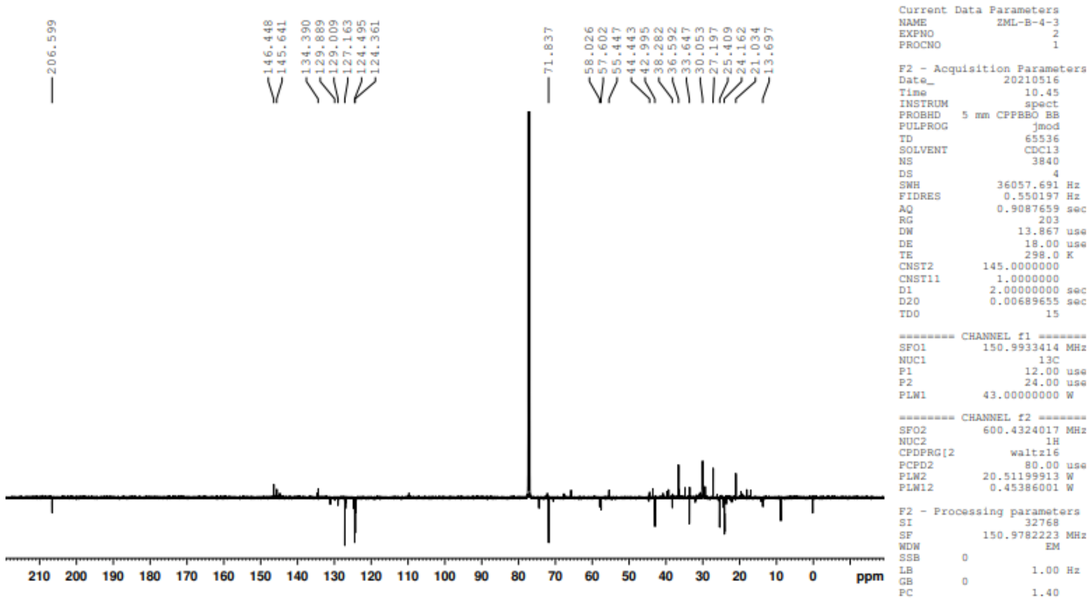

Figure S8 ^{13}C-APT (150 MHz, $CDCL_3$) spectrum of **2**

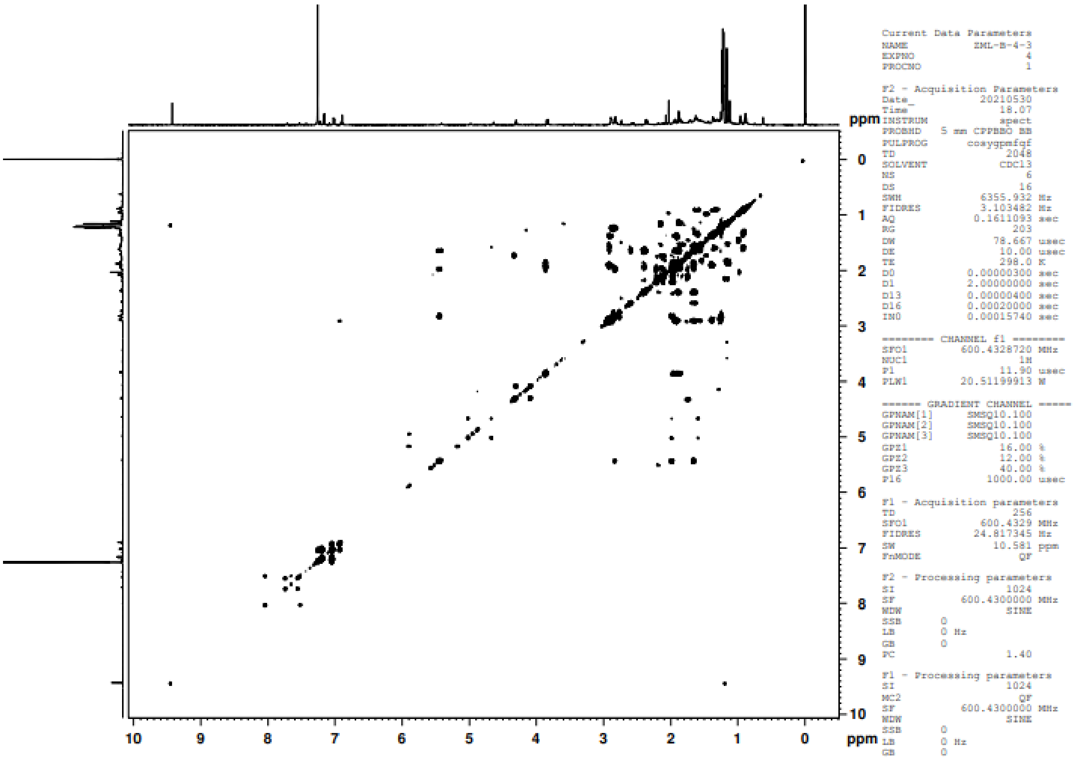

Figure S9 ^{1}H-^{1}H COSY ($CDCL_3$) spectrum of **2**

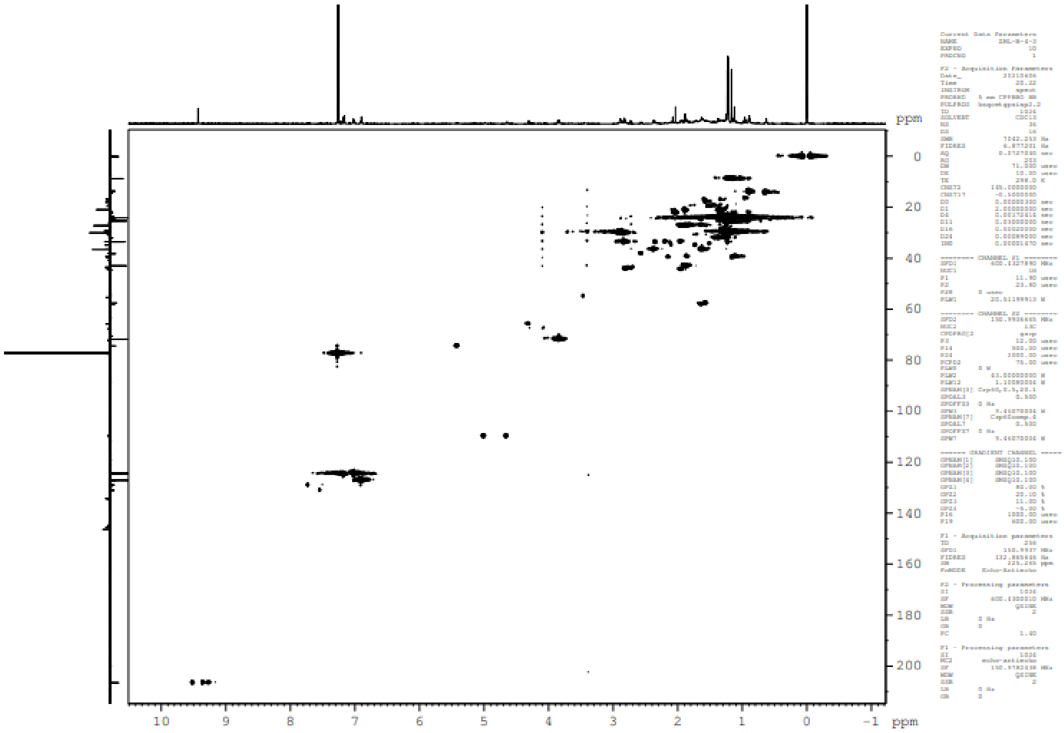

Figure S10 HSQC ($CDCL_3$) spectrum of **2**

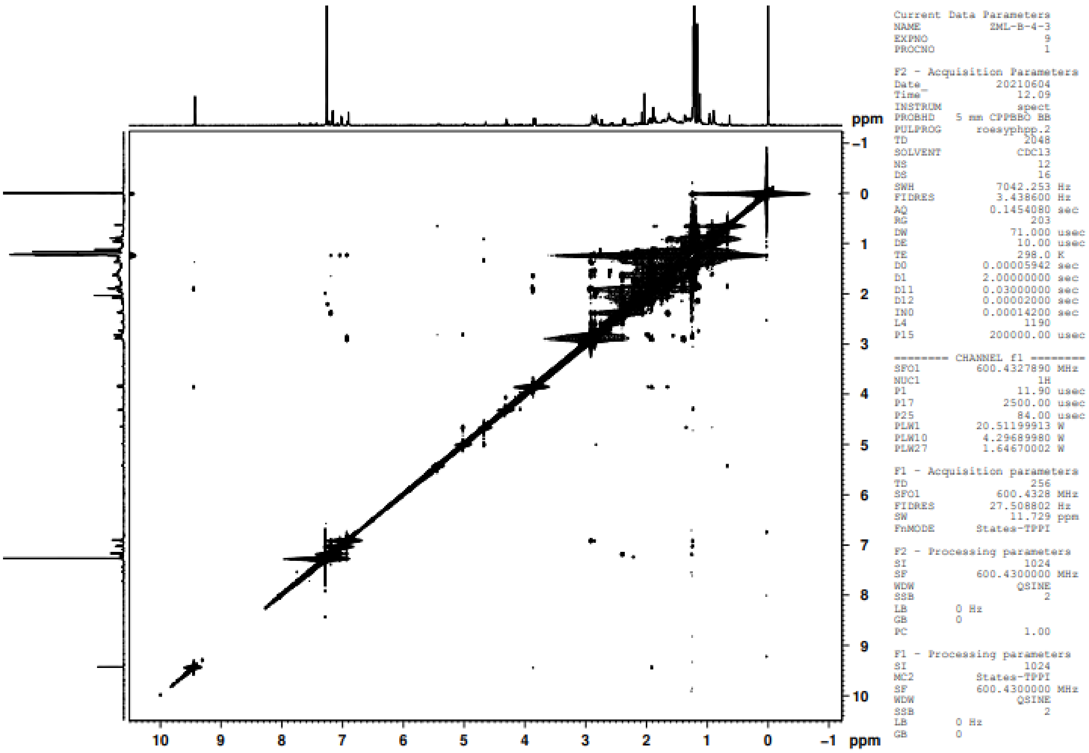

Figure S11 NOESY ($CDCL_3$) spectrum of **2**

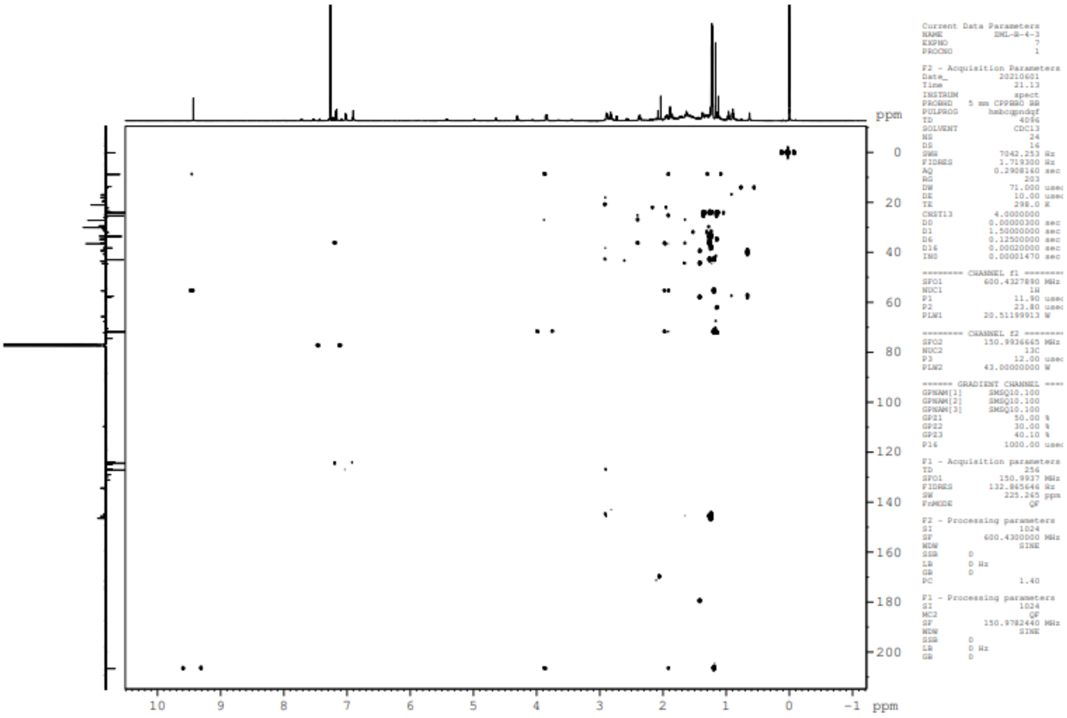

Figure S12 HMBC ($CDCL_3$) spectrum of **2**

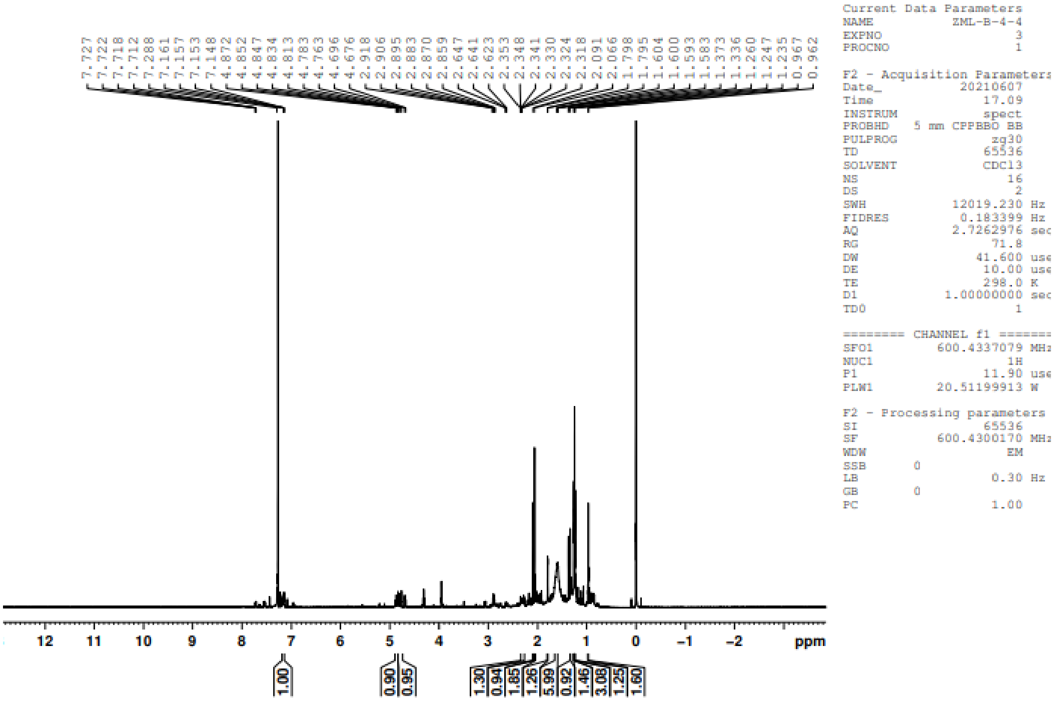

Figure S13 ^{1}H-NMR (600 MHz, CDCL$_3$) spectrum of **3**

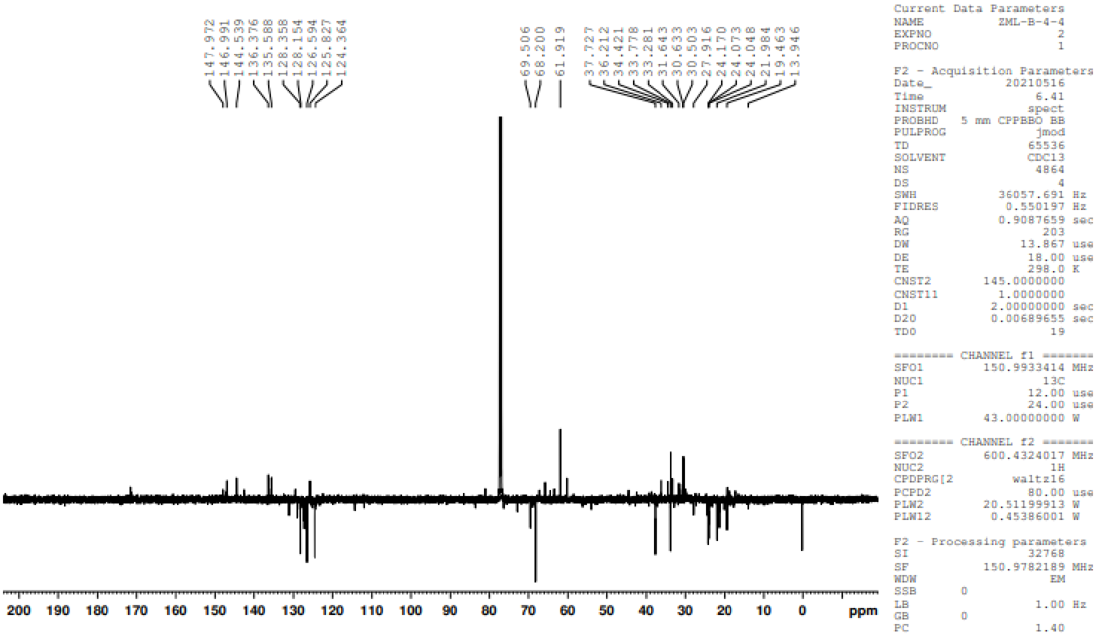

Figure S14 ^{13}C-APT (150 MHz, CDCL$_3$) spectrum of **3**

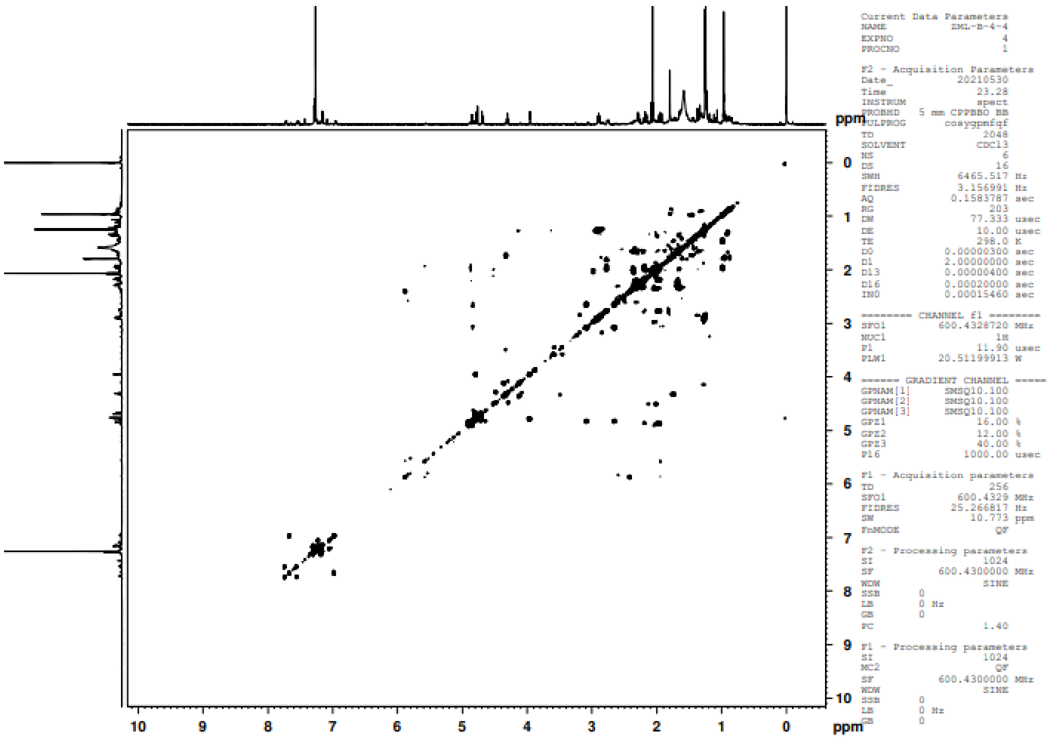

Figure S15 ^{1}H-^{1}H COSY (CDCL$_3$) spectrum of **3**

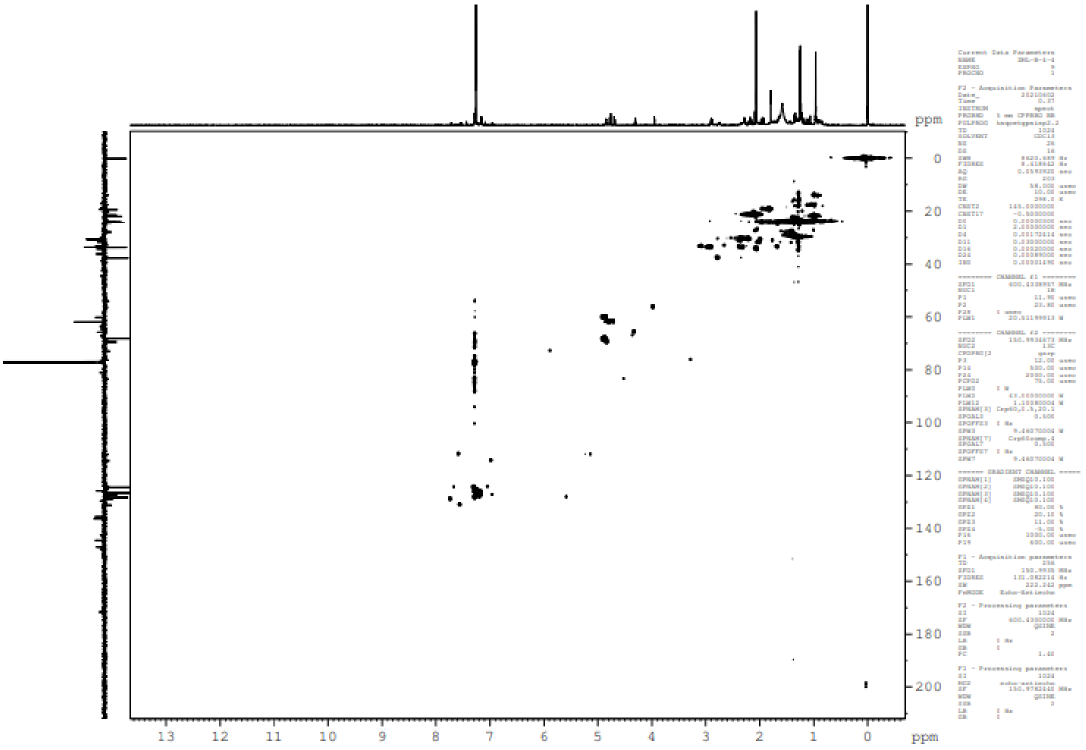

Figure S16 HSQC (CDCL$_3$) spectrum of **3**

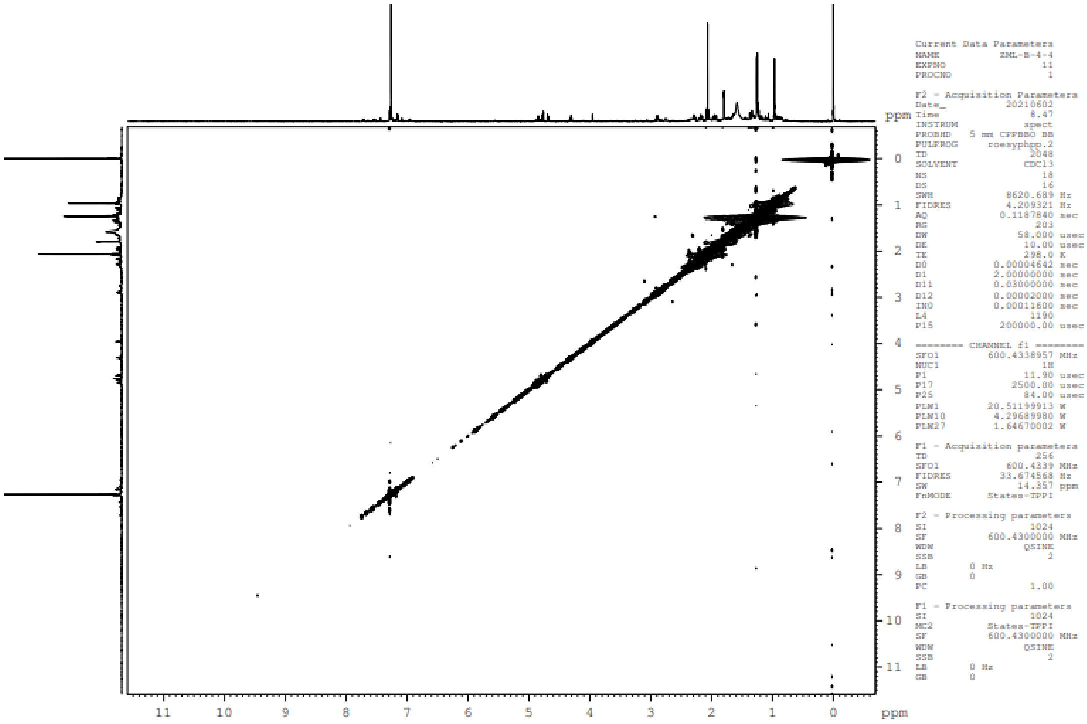

Figure S17 NOESY ($CDCL_3$) spectrum of **3**

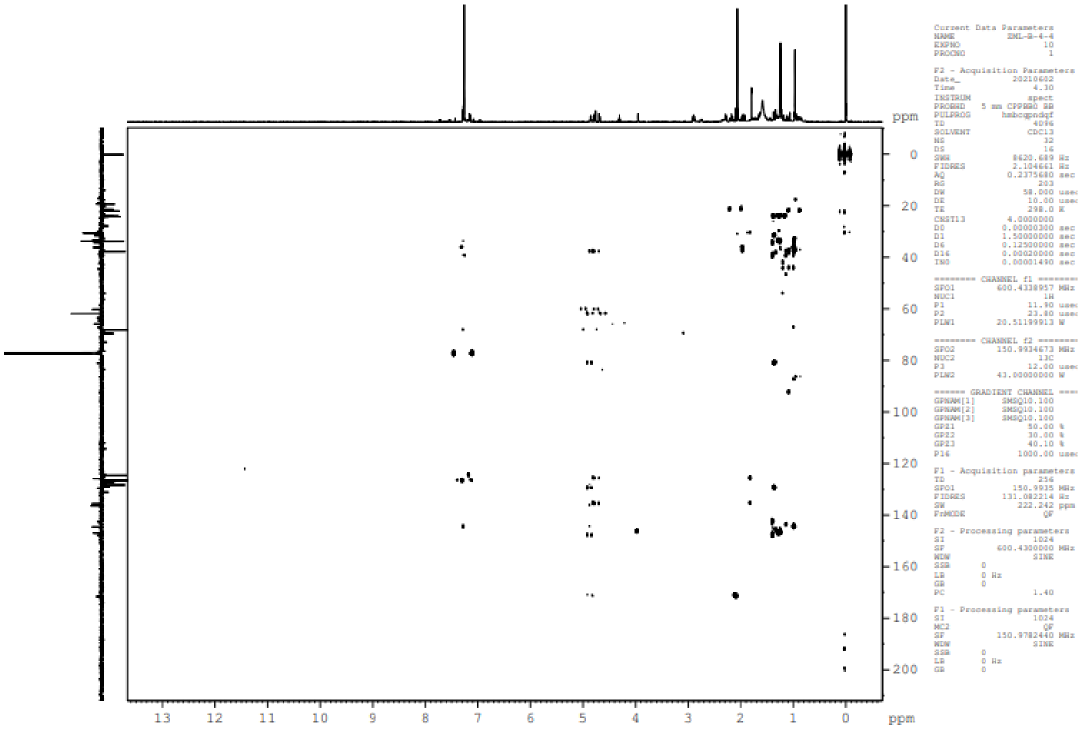

Figure S18 HMBC ($CDCL_3$) spectrum of **3**

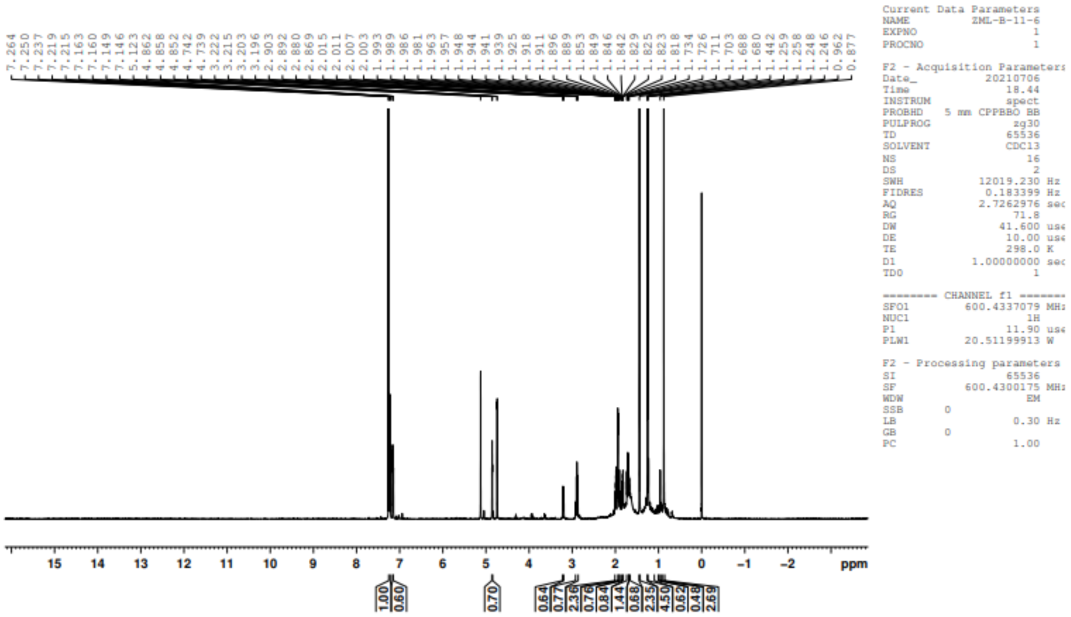

Figure S19 ^{1}H-NMR (600 MHz, CDCL$_3$) spectrum of **4**

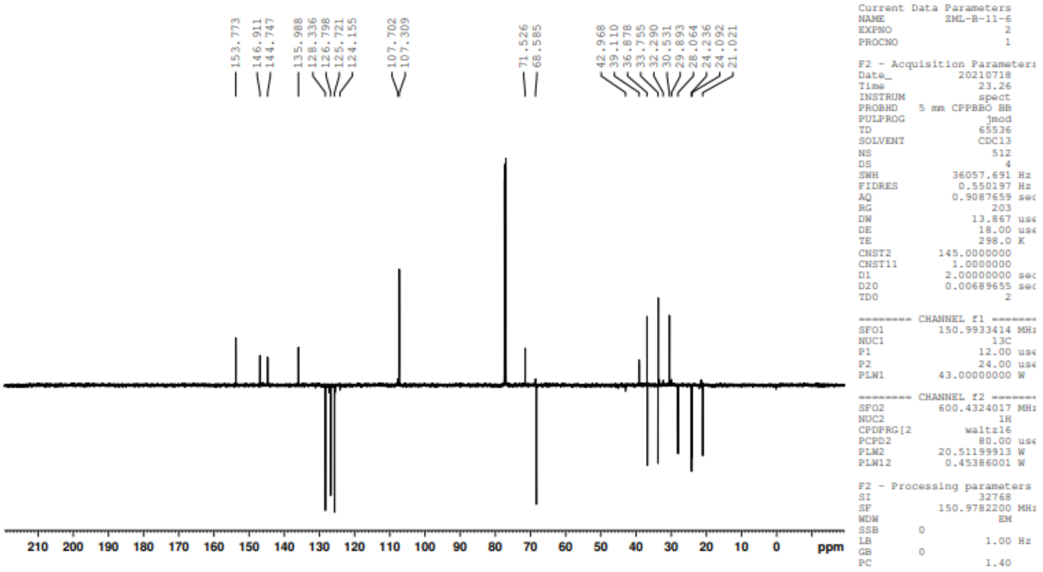

Figure S20 ^{13}C-APT (150 MHz, CDCL$_3$) spectrum of **4**

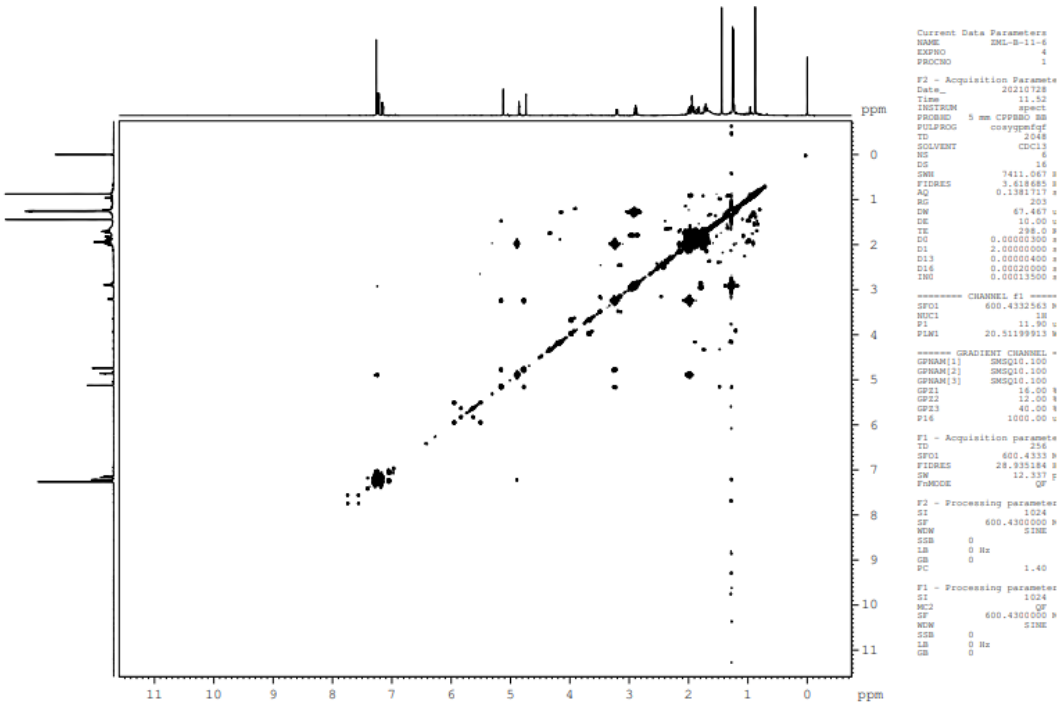

Figure S21 ^{1}H-^{1}H COSY (CDCL$_3$) spectrum of **4**

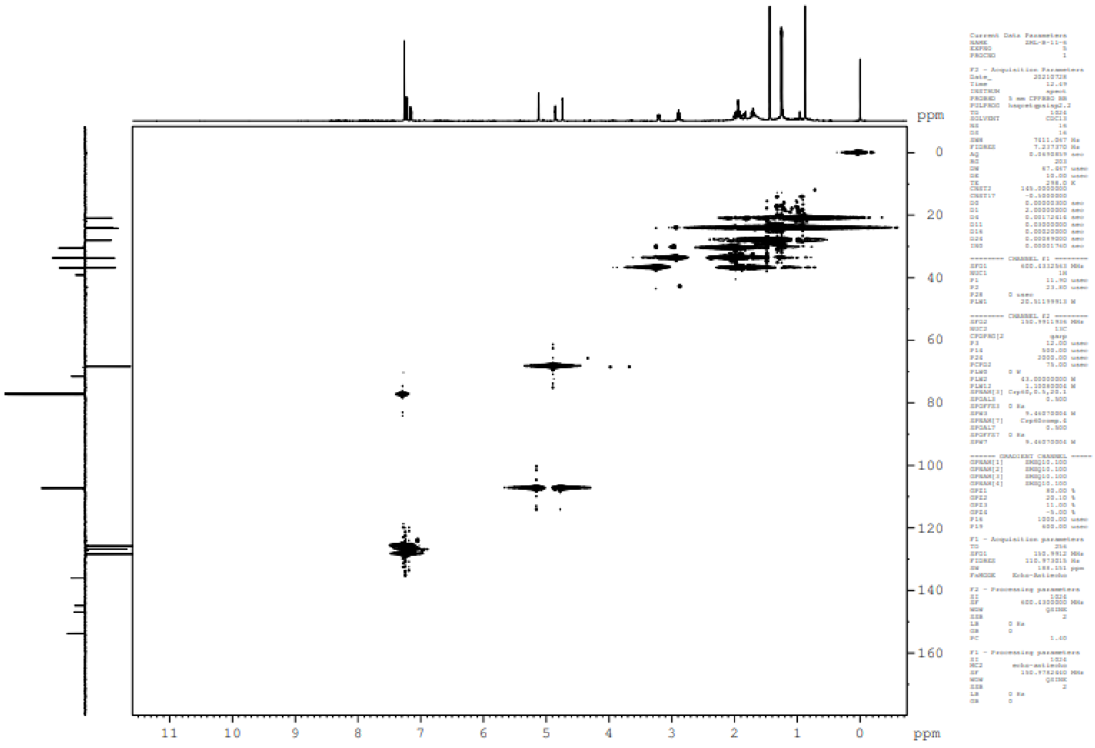

Figure S22 HSQC (CDCL$_3$) spectrum of **4**

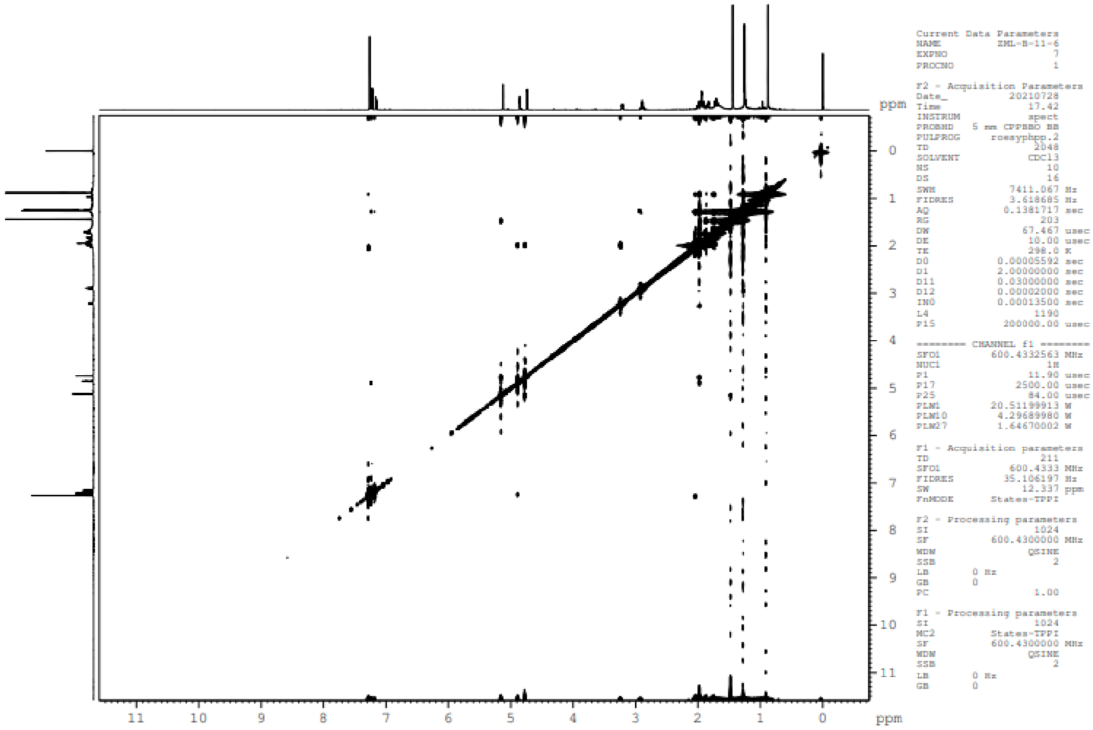

Figure S23 NOESY (CDCL$_3$) spectrum of **4**

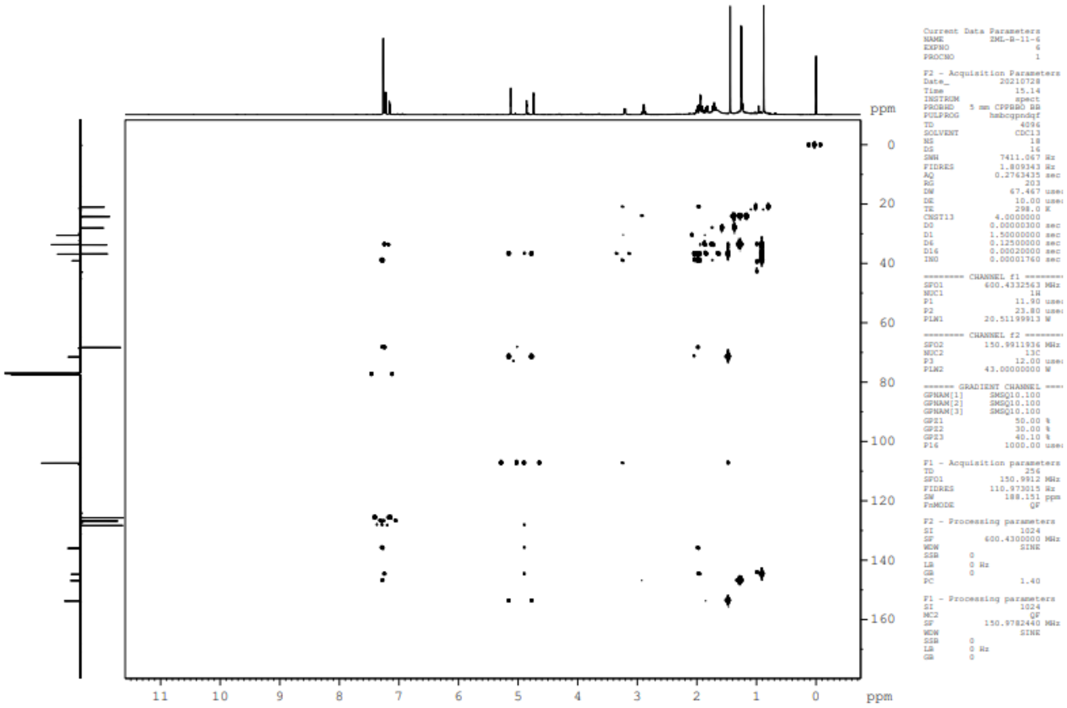

Figure S24 HMBC (CDCL$_3$) spectrum of **4**

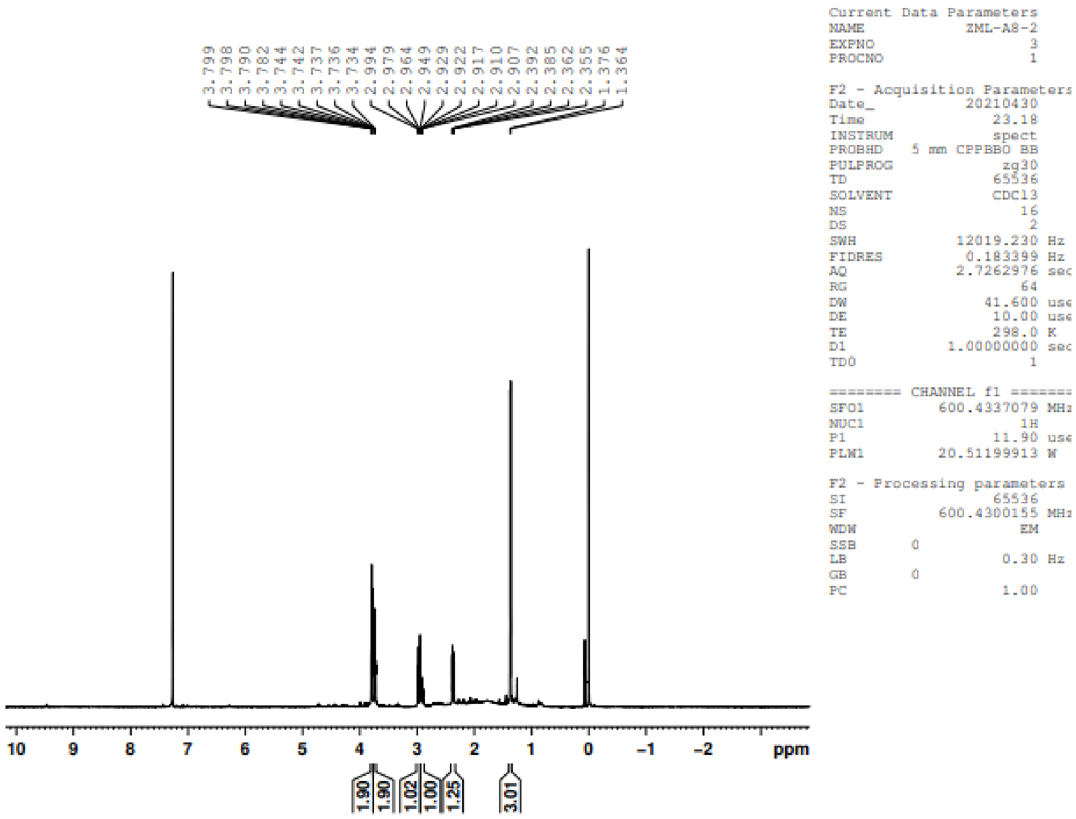

Figure S25 ^{1}H-NMR (600 MHz, CDCL$_3$) spectrum of **5**

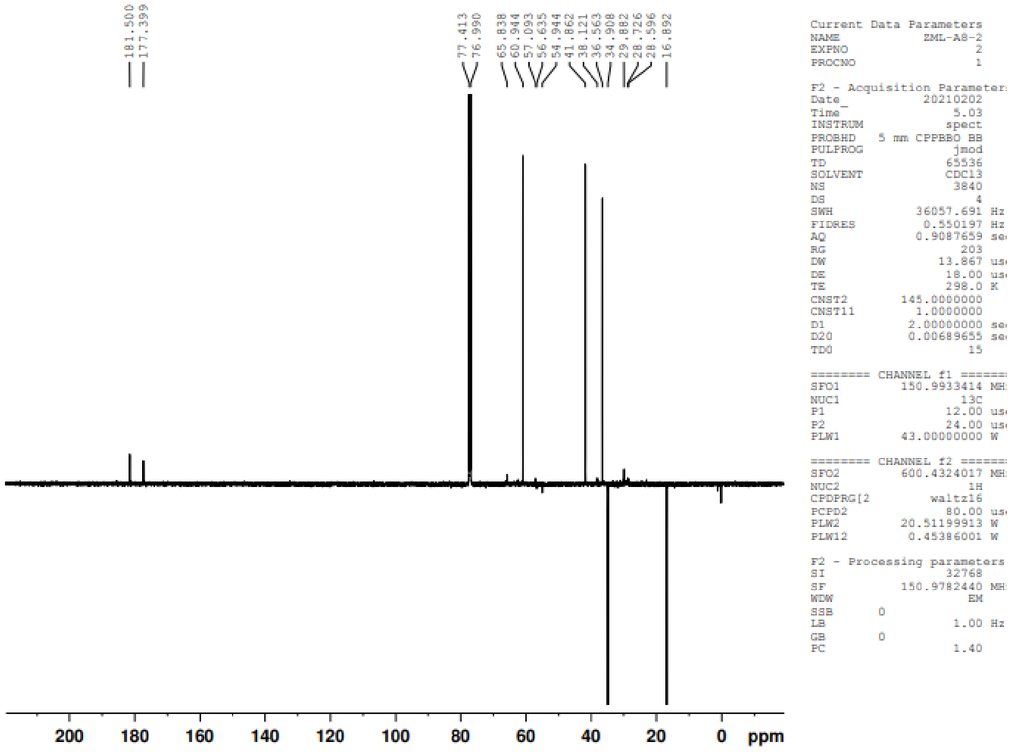

Figure S26 ^{13}C-APT (150 MHz, CDCL$_3$) spectrum of **5**

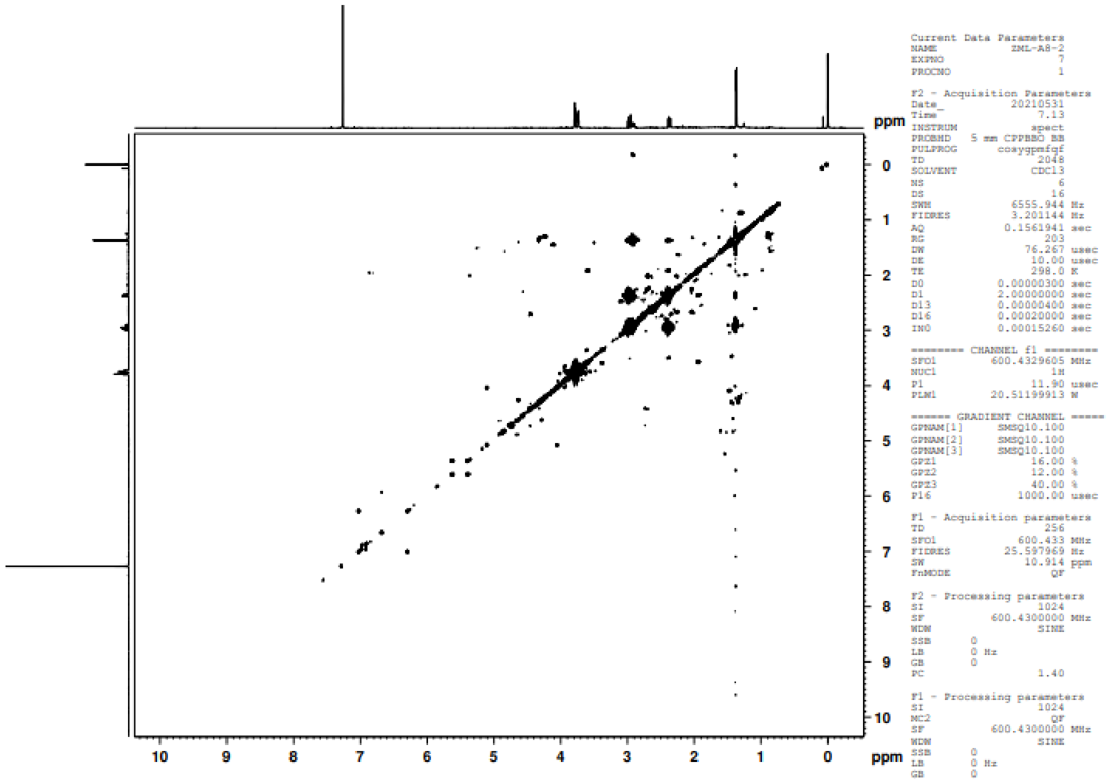

Figure S27 ^{1}H-^{1}H COSY ($CDCL_3$) spectrum of **5**

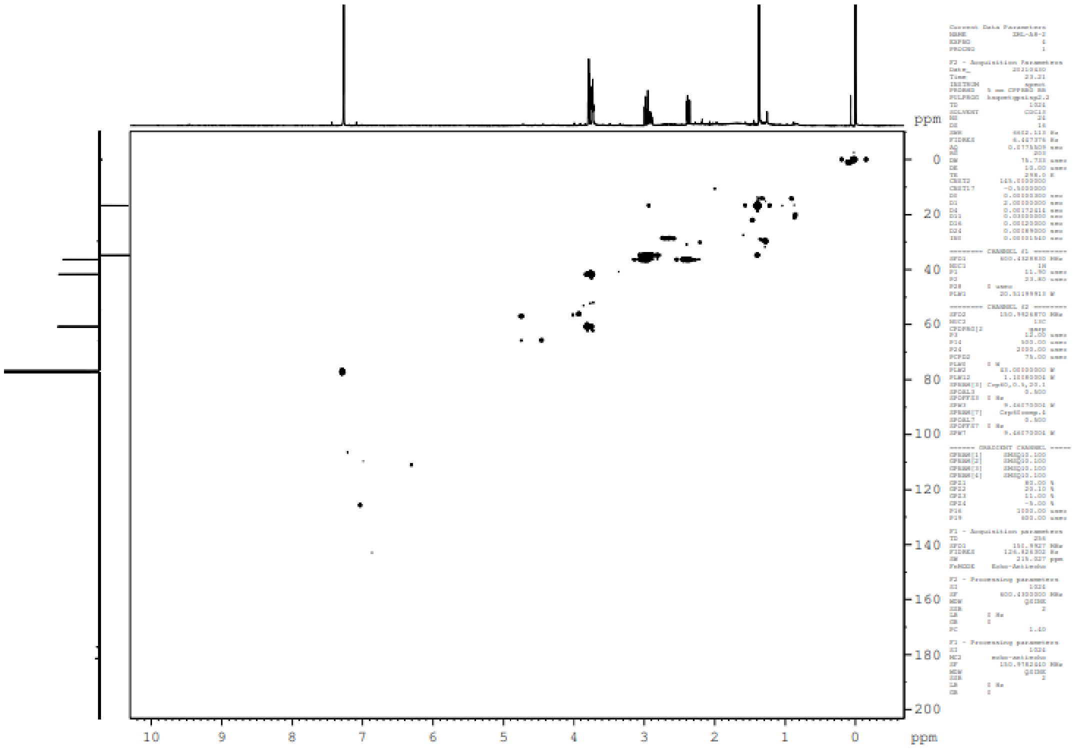

Figure S28 HSQC ($CDCL_3$) spectrum of **5**

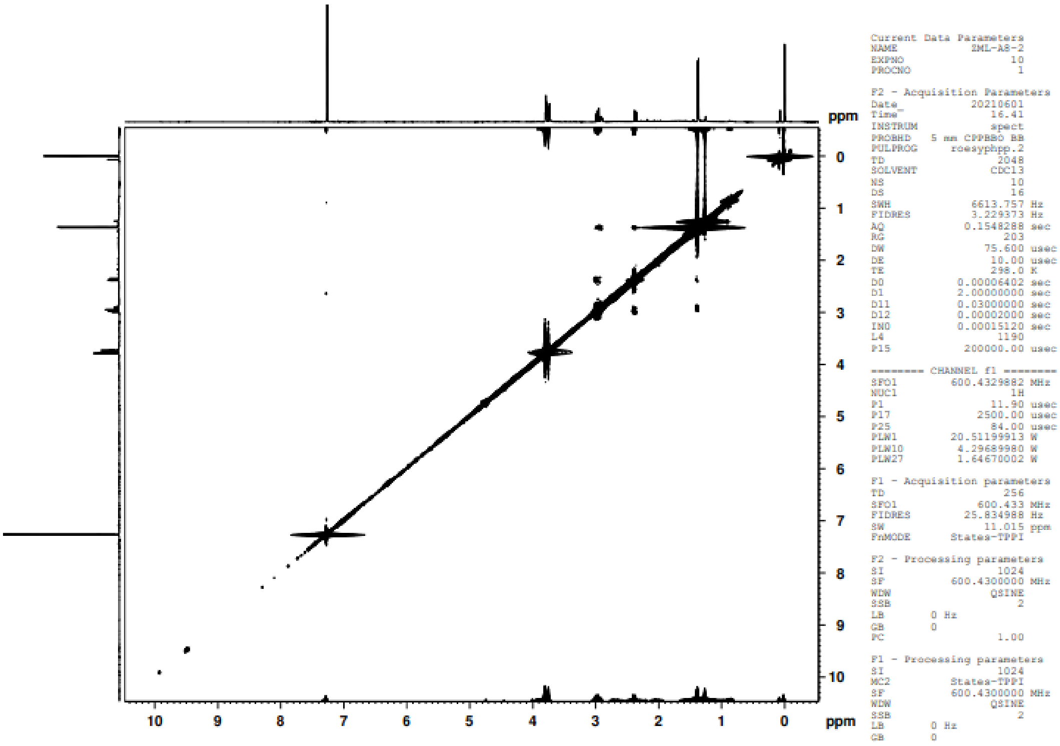

Figure S29 NOESY ($CDCL_3$) spectrum of **5**

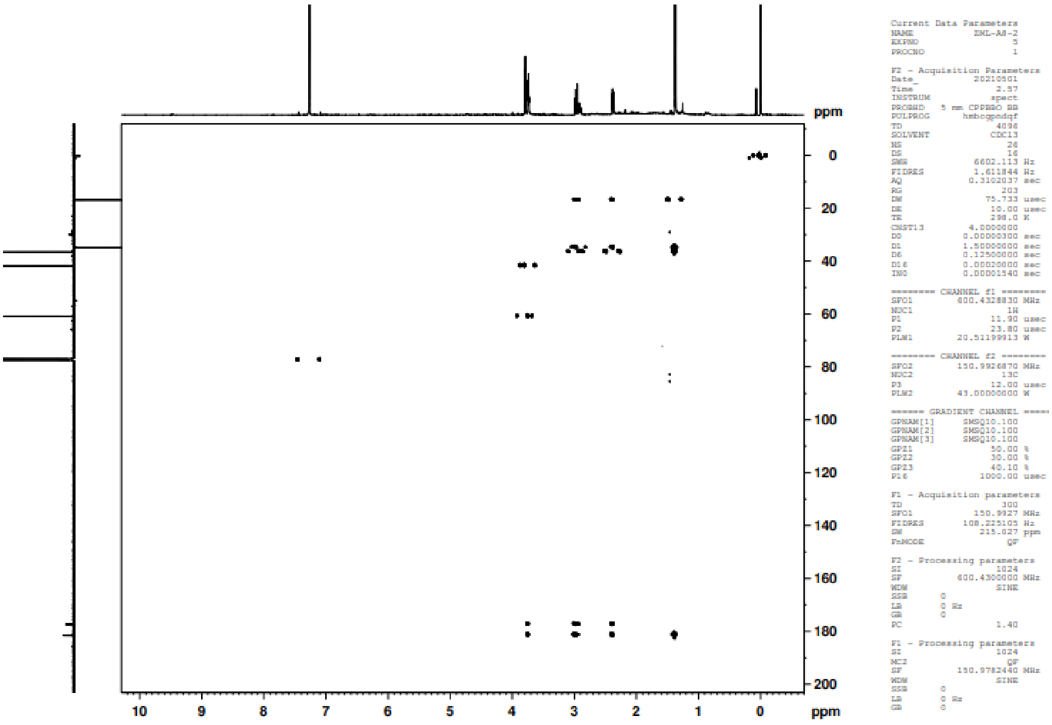

Figure S30 HMBC ($CDCL_3$) spectrum of **5**

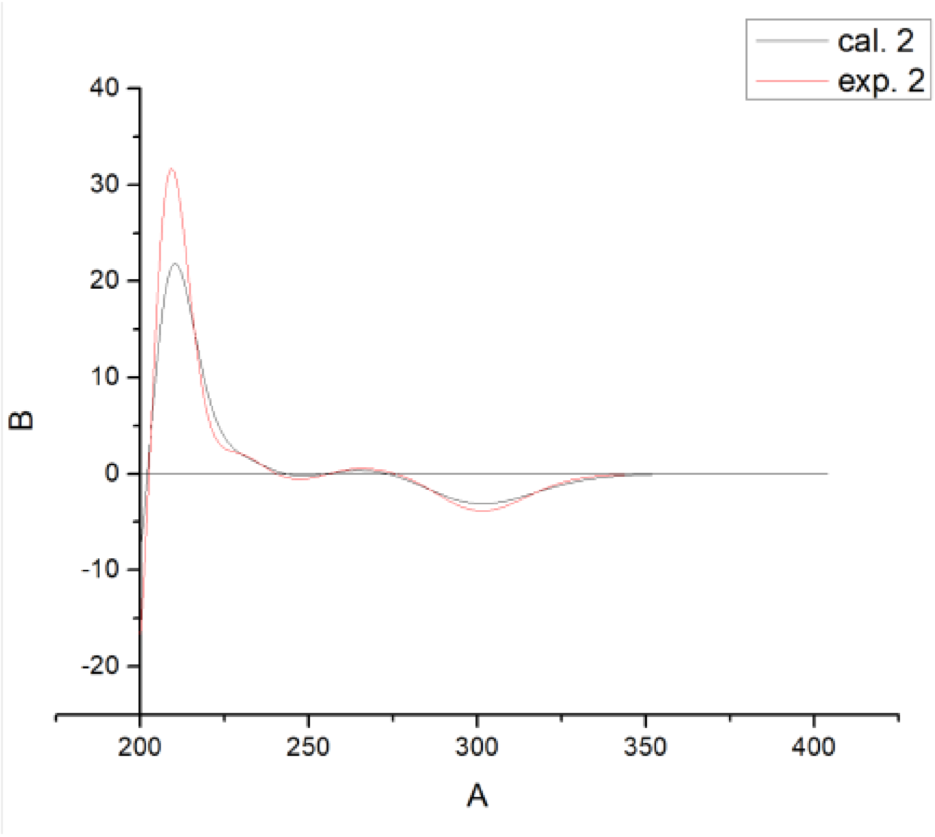

Figure S31 Experimental and calculated ECD spectra of **2**

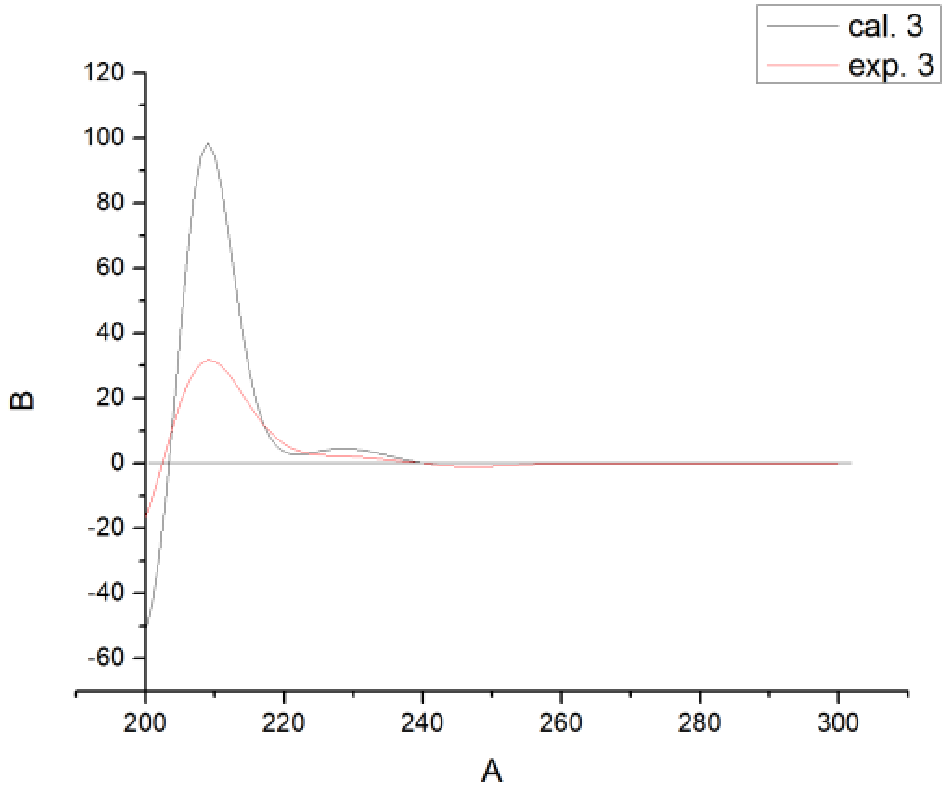

Figure S32 Experimental and calculated ECD spectra of **3**

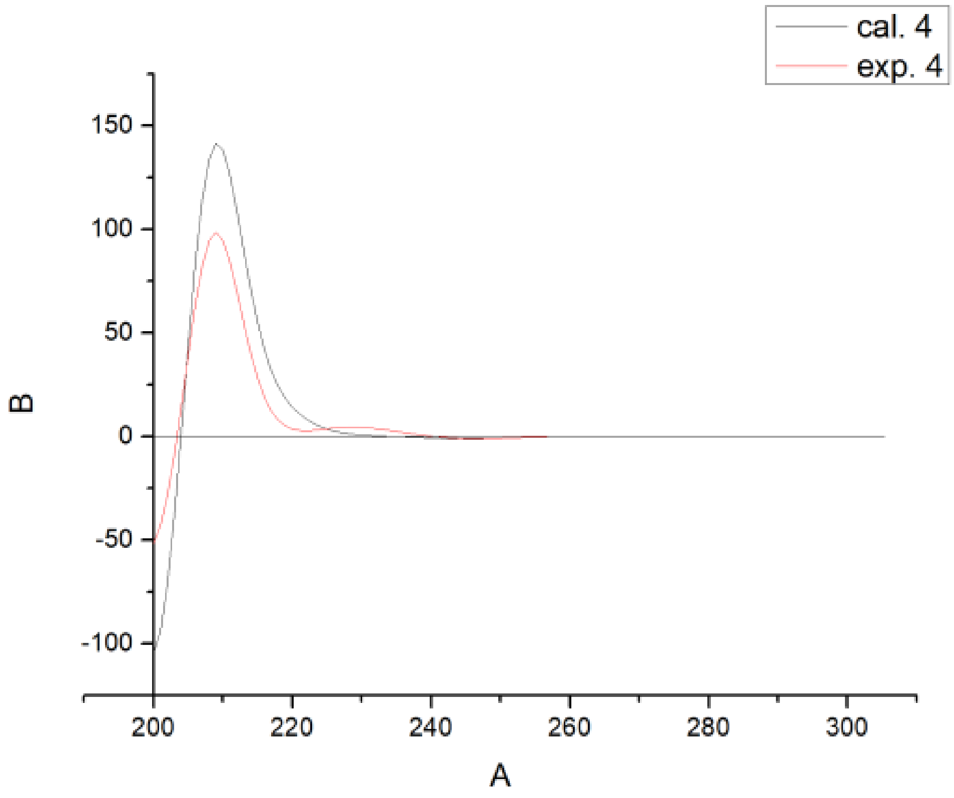

Figure S33 Experimental and calculated ECD spectra of **4**

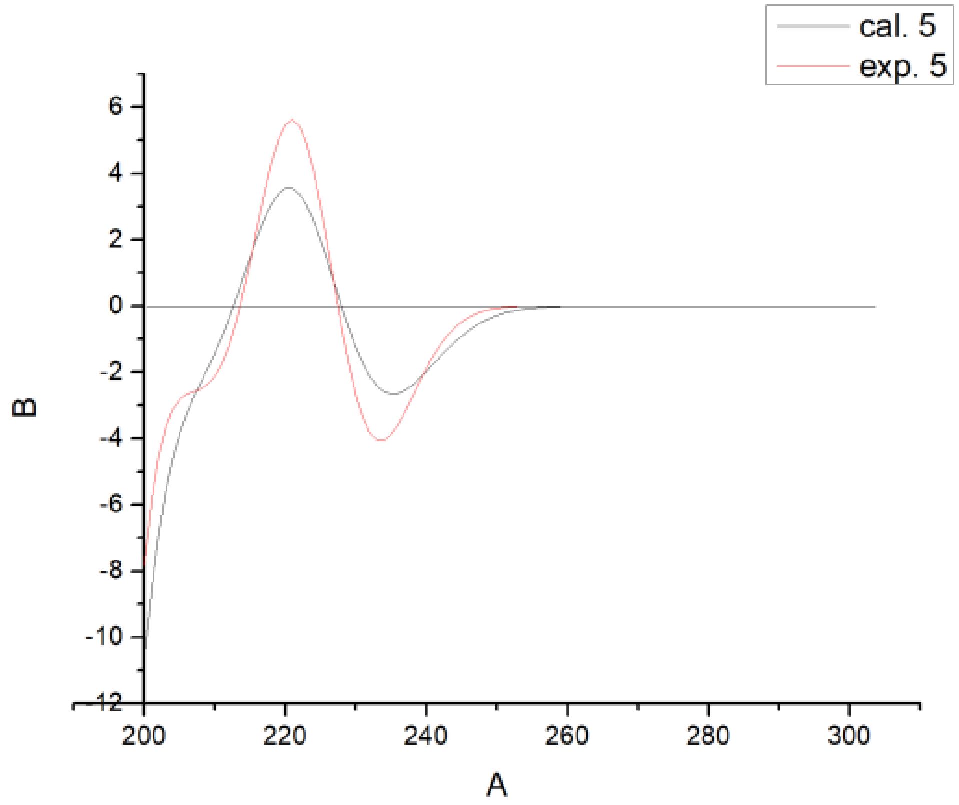

Figure S34 Experimental and calculated ECD spectra of **5**
